# Multifunctional Y_2_O_3_-Modified Borotellurite Bioactive Glasses for Bone Tissue Engineering Applications

**DOI:** 10.3390/jfb17050240

**Published:** 2026-05-09

**Authors:** Esmanur Oruc Ulas, Bulent Aktas, Abuzer Acikgoz, Serife Yalcin, Hatice Gumushan Aktas, Ebru Uyar, Zeynep Celik

**Affiliations:** 1 Department of Mechanical Engineering, Harran University, Şanlıurfa 63050, Türkiye; 2 Department of Physics, Harran University, Şanlıurfa 63050, Türkiye; serifeyalcin@harran.edu.tr; 3 Department of Biology, Harran University, Şanlıurfa 63050, Türkiye; haticeaktas@harran.edu.tr (H.G.A.); ebruuyar@harran.edu.tr (E.U.); zeynepcelik@harran.edu.tr (Z.C.)

**Keywords:** borotellurite bioactive glass, Y_2_O_3_ doping, bone tissue engineering, cytocompatibility, antibacterial activity

## Abstract

Developing bioactive glasses that simultaneously provide mechanical reliability, cytocompatibility, controlled ion release, and antibacterial functionality remains a major challenge in bone tissue engineering. In this study, borotellurite-based bioactive glasses with the composition (45 − x)TeO_2_–20Na_2_O–10CaO–15P_2_O_5_–10B_2_O_3_–xY_2_O_3_ (x = 0–7 mol.%) were designed to elucidate the role of Y_2_O_3_ in governing composition–structure–property relationships. Structural, thermal, mechanical, ion-release, bioactivity, cytocompatibility, cell-adhesion, and antibacterial properties were systematically evaluated, and the most promising composition was further modified by silver surface coating. Y_2_O_3_ incorporation markedly enhanced thermal stability, hardness, and fracture resistance, with hardness reaching 4.317 GPa at 7 mol.%, while the highest compressive strength was achieved at 1 mol.% Y_2_O_3_ (67.97 MPa). Importantly, Y_2_O_3_ regulated dissolution behavior and mitigated the severe long-term cytotoxicity of the undoped glass, maintaining all doped compositions above the ISO 10993-5 threshold after 30 days. Higher Y_2_O_3_ contents also promoted osteoblast adhesion and facilitated bioactive surface layer formation following SBF immersion. No detectable *E. coli* adhesion was observed, whereas the TBY3 composition exhibited the lowest *S. aureus* adhesion, further improved by silver coating. These results demonstrate Y_2_O_3_ as an effective multifunctional modifier for engineering mechanically robust, biologically favorable, and antibacterial borotellurite bioactive glasses for bone repair.

## 1. Introduction

Bone defects caused by trauma, tumor resection, congenital abnormalities, and age-related disorders such as osteoporosis continue to represent a substantial clinical burden worldwide, with millions of bone grafting procedures performed annually [1]. When the defect size exceeds the intrinsic regenerative capacity of bone, biomaterial-based substitutes are required to provide structural support, facilitate cellular infiltration, and promote new tissue formation [2]. Among the available candidates, bioactive glasses have emerged as one of the most versatile classes of bone-repair biomaterials because of their ability to bond directly to living bone through the formation of a hydroxycarbonate apatite layer, release biologically active ions that stimulate osteogenesis, and accommodate broad compositional modifications for enhanced therapeutic functionality [3,4]. Since the introduction of 45S5 Bioglass, the field has evolved from conventional silicate formulations toward increasingly complex multifunctional systems designed to meet the mechanical, biological, and antimicrobial requirements of clinically relevant bone regeneration [2,4].

Despite their outstanding bioactivity, conventional silicate-based bioactive glasses remain limited by relatively poor mechanical reliability, especially low fracture toughness and insufficient resistance under load-bearing conditions [5,6]. These shortcomings have prompted extensive efforts to identify alternative glass formers capable of improving structural performance without compromising biological activity. In this respect, tellurium dioxide (TeO_2_) is a particularly interesting network former because of its high chemical durability, structural flexibility, and the coexistence of [TeO_4_] [7,8] and [TeO_3_] units arising from the lone-pair electrons of Te^4+^ [9,10]. These features provide tellurite glasses with high polarizability and relatively strong network connectivity, which may enable more controlled degradation behavior than that of highly soluble phosphate-dominated systems [11,12]. The incorporation of boron trioxide (B_2_O_3_) as a co-network former may further strengthen this platform by enhancing thermal stability and network cross-linking through the formation of [BO_4_] units. In addition, boron-containing dissolution products have been associated with beneficial effects on bone-related cellular functions, including mineralized nodule formation and the expression of osteogenic markers, when released within appropriate concentration windows [11,13]. However, despite these promising attributes, borotellurite glasses remain insufficiently explored for bone tissue engineering, particularly in terms of long-term dissolution behavior, cytocompatibility, and antibacterial performance [14].

Rare-earth oxide incorporation has recently gained attention as a powerful strategy for tuning the structure and functional behavior of multicomponent glass systems [15,16]. Among these oxides, Y_2_O_3_ is especially attractive because Y^3+^ ions can reinforce the glass network through strong Y–O interactions, increase cross-link density, and improve resistance to deformation and chemical degradation [17,18]. Previous computational and experimental studies have suggested that yttrium incorporation can influence local network connectivity, phosphate environments, and calcium distribution, thereby potentially affecting mineralization-related processes [7,19]. In addition, Y_2_O_3_-containing glass and ceramic materials have generally demonstrated acceptable biocompatibility at relevant concentrations, supporting their potential use in implantable biomedical systems [20,21,22]. It should also be acknowledged that ion-mediated compositional modification is an established strategy in bioactive glass and glass–ceramic research. Various therapeutic or functional ions, including Sr, Zn, Ce, La, and Ag, have been incorporated into different glass matrices to improve bioactivity, osteogenic response, antibacterial performance, and physicochemical stability. Recent studies have further demonstrated that processing conditions and compositional design strongly influence the biological performance of phosphate-based mesoporous glass–ceramic nanoparticles for bone regeneration [23], while Ag incorporation has been shown to enhance the osteogenic and antibacterial functionality of bioactive spherical glass–ceramic nanoparticles [24]. However, most of these approaches have focused on silicate-, phosphate-, calcium phosphate-, or glass–ceramic nanoparticle-based systems. In comparison, TeO_2_-rich borotellurite bioactive glasses remain much less explored, particularly in terms of how Y_2_O_3_ incorporation affects long-term tellurium release, cytocompatibility, mechanical reliability, bioactive surface reactions, and antibacterial behavior. Therefore, the novelty of the present study is not based solely on the general concept of oxide doping, but rather on clarifying the multifunctional role of Y_2_O_3_ within a TeO_2_-rich borotellurite bioactive glass matrix and evaluating its combined effects on structural, mechanical, dissolution, biological, and antibacterial responses. Nevertheless, the structural and biological role of Y_2_O_3_ in TeO_2_-rich glass matrices remains largely unexplored. This gap is important because, in tellurite-based systems, compositional stabilization may have consequences far beyond mechanical reinforcement and may directly influence the release of biologically critical ions during prolonged degradation.

In parallel with the need for mechanically and biologically optimized bone substitutes, implant-associated infection remains a major clinical challenge in orthopedic and reconstructive applications. Pathogens such as *Staphylococcus aureus* and *Escherichia coli* can rapidly adhere to implanted surfaces and form biofilms that are highly tolerant to conventional antibiotic therapy [25,26,27]. For this reason, silver-based surface functionalization has become one of the most widely investigated strategies for imparting antibacterial activity to implant-related biomaterials. Ag^+^ ions exhibit broad-spectrum antimicrobial effects through multiple mechanisms, including membrane disruption, oxidative stress induction, impairment of enzymatic respiration, and interactions with nucleic acids and proteins [28,29]. When introduced as a surface coating, silver can provide localized antibacterial protection while minimizing systemic exposure, and previous studies have shown that Ag-functionalized ceramic and glass-derived coatings can effectively reduce bacterial adhesion and biofilm formation [30,31]. Thus, integrating an optimized bioactive glass composition with silver surface functionalization offers a promising route toward multifunctional bone biomaterials capable of combining structural support, cytocompatibility, and infection resistance.

A further concern specific to tellurite-containing systems is the potential cytotoxicity associated with prolonged release of tellurite species. Although low concentrations of tellurite may contribute to antibacterial action, excessive or sustained ion release can adversely affect mammalian cells by inducing oxidative stress and mitochondrial dysfunction [32]. This issue is particularly relevant in the context of long-term bone-contact applications, where maintaining osteoblast viability is essential. Therefore, a key unresolved question is whether Y_2_O_3_ incorporation can stabilize TeO_2_-rich borotellurite glass networks sufficiently to suppress harmful long-term ion release while preserving the beneficial structural and biological features of the system. Clarifying this balance is essential for assessing the translational potential of tellurite-based bioactive glasses.

Based on this rationale, the present study developed a novel borotellurite bioactive glass system with the composition 45TeO_2_–20Na_2_O–10CaO–15P_2_O_5_–10B_2_O_3_, partially modified with 1–7 mol.% Y_2_O_3_ through substitution of TeO_2_. The effects of Y_2_O_3_ incorporation on glass structure, thermal behavior, physical characteristics, mechanical performance, ion release, in vitro bioactivity, cytocompatibility, osteoblast adhesion, and antibacterial behavior were systematically investigated. In addition, the most promising composition was further functionalized with a silver coating to enhance antibacterial efficacy. By integrating compositional optimization with surface modification, this work aims to establish clear composition–structure–property relationships in Y_2_O_3_-modified borotellurite bioactive glasses and to demonstrate that Y_2_O_3_ acts not only as a reinforcing additive, but also as a critical biocompatibility-enabling modifier for bone tissue engineering applications.

## 2. Materials and Methods

### 2.1. Synthesis and Production of Bioactive Glasses

High-purity H_3_BO_3_ (99.9%, Merck KGaA, Darmstadt, Germany), Na_2_CO_3_ (99.9%, Merck KGaA, Darmstadt, Germany), TeO_2_ (99.9%, Nanografi Nano Technology, Ankara, Türkiye), P_2_O_5_ (>98%, Nanografi Nano Technology, Ankara, Türkiye), CaO (>99.95%, Nanografi Nano Technology, Ankara, Türkiye), and Y_2_O_3_ (99.99%, Nanografi Nano Technology, Ankara, Türkiye) were used as raw materials for the preparation of the glass specimens. The general properties of these starting materials are presented in Table 1.

The nominal glass composition investigated in this study was (45 − x)TeO_2_–20Na_2_O–10CaO–15P_2_O_5_–10B_2_O_3_–xY_2_O_3_, which was introduced through equimolar substitution of TeO_2_ to maintain total oxide content at 100 mol.%. The resulting five compositions are designated TB (x = 0), TBY1 (x = 1), TBY3 (x = 3), TBY5 (x = 5), and TBY7 (x = 7), and their compositions are presented in Table 2. The powder mixtures (Batch masses of 10 g per composition) were weighed on an analytical balance with a readability of 0.00001 g (Radwag AS 220.R2).

Each powder mixture was homogenized by mechanical stirring at 600 rpm for 15 min. The mixtures were melted in a high-temperature furnace (MSE 1600, MSE Tecnology, Kocaeli, Türkiye) using alumina crucibles under ambient atmospheric conditions. Given the known volatility of P_2_O_5_ at elevated temperatures, covered crucibles were used throughout the melting stage to minimize evaporative loss; the actual oxide contents were assumed to correspond to the nominal batch compositions. The melting procedure was carried out in three stages: the furnace temperature was first raised to 200 °C within 40 min and held for 2 h to ensure complete dehydration of H_3_BO_3_; it was then raised to 600 °C within 80 min and held for 120 min to facilitate decarbonation of Na_2_CO_3_; finally, the temperature was raised to 1150 °C within 70 min to complete the melting process. The prepared melt was quickly poured into a graphite mold preheated to 280 °C to produce cylindrical glass samples, 5 mm in height and 2 mm in diameter, for compression testing, and disc-shaped specimens approximately 2 mm thick for all other characterization procedures. To relieve thermal residual stress, all samples were annealed at 300 °C for 3 h and subsequently cooled slowly to room temperature. The annealing temperature was selected based on the glass transition temperature of the base composition [33].

### 2.2. Determination of Physical, Structural, Optical, and Thermal Properties

The amorphous nature and phase composition of the as-cast glasses and Simulated body fluid (SBF)-treated surfaces were analyzed by X-ray diffraction (XRD) using a Rigaku D/Max-2000 diffractometer (Rigaku Corporation, Tokyo, Japan) equipped with CuKα radiation (λ = 1.5405 Å). Diffraction patterns were recorded over a 2θ range of 5–70° with a step size of 0.02° and a counting time of 1 s per step. Phase identification was performed using the ICDD PDF-4+ database, 2023 release. In the SBF-treated samples, the formation of a hydroxyapatite layer was assessed by monitoring the characteristic reflections at 2θ ≈ 25.9° (002), 31.7° (211), and 32.9° (300).

Fourier-transform infrared (FTIR) spectra were recorded using a Bruker Hyperion 3000 FTIR spectrometer equipped with an attenuated total reflectance (ATR) accessory (Bruker Optics GmbH, Ettlingen, Germany) within the range of 500–4000 cm^−1^. Measurements were performed with a spectral resolution of 4 cm^−1^ and 64 scans per sample. The acquired spectra were baseline-corrected and normalized prior to analysis, and band assignments were interpreted based on reported vibrational modes of TeO_2_–B_2_O_3_–P_2_O_5_ glass systems.

The density (ρ) of the glass samples (Radwag AS 220.R2) was determined using Archimedes principle with a Radwag analytical balance equipped with a density measurement kit, employing distilled water (ρ = 0.9982 g/cm^3^ at 20 °C) as the immersion medium. At least three independent measurements were conducted for each composition, and the average values were used for further calculations. Based on the experimental density values, key structural parameters including molar volume (*V_m_*), atomic packing density (*V_t_*), oxygen packing density (*OPD*), molar oxygen volume (*MOV*), average cross-linking density (*ñ_c_*), bond density (*n_b_*), and dissociation energy (*G_t_*) were calculated using the equations provided in Table 3. The dissociation energy values for the constituent oxides were taken from the literature of Inaba et al. [34].

UV–Vis optical transmittance spectra were measured at room temperature using a PerkinElmer Lambda 25 UV–Vis spectrophotometer (PerkinElmer Inc., Waltham, MA, USA) over the wavelength range of 300–1000 nm. The optical bandgap energy (E_g_) was determined using the Tauc method by extrapolating the linear region of the (αhν)^2^ versus photon energy (hν) plot to the energy axis, where the absorption coefficient (α) was calculated based on the Beer–Lambert law. In addition to the bandgap energy, key optical parameters, including the refractive index (*n*), extinction coefficient (*k*), metallization criterion (*M*), optical electronegativity, optical basicity (*Λ*), dielectric constant (*ε*), and linear dielectric susceptibility (*χ*), were calculated from the absorption data using the relationships summarized in Table 4.

Differential thermal analysis (DTA) was performed using a DTA/TG thermal analysis system (Shimadzu DTG-60H) from room temperature to 800 °C at a heating rate of 10 °C/min under a nitrogen atmosphere, using α-Al_2_O_3_ as the reference material. The glass transition temperature (T_g_), crystallization onset temperature (T_c_), and first and second melting temperatures (T_m1_, T_m2_) were determined from the inflection point of the endothermic baseline shift and the onset of exothermic and endothermic peaks, respectively. The Hrubrek glass stability parameter (ΔT = T_c_ − T_g_) was calculated as a quantitative measure of resistance to devitrification during thermal processing [35].

### 2.3. Determination of Elastic and Mechanical Properties

Prior to mechanical characterization, all samples were ground and polished to a mirror-like finish using progressively finer SiC abrasive papers, followed by final polishing with 3 μm and 1 μm diamond suspensions to ensure a smooth and defect-free surface for indentation measurements.

Vickers microhardness was measured at room temperature using an AOB Lab micro Vickers hardness tester (AOB Lab, Istanbul, Türkiye) under a 1000 g load applied for 5 s. At least five indentations were performed for each composition, and the average diagonal length was used to calculate the hardness (*H_v_*) according to Equation (1).

(1) $$H_{v}=\frac{1.854P}{d^{2}}$$

where H_v_ is the Vickers hardness (kg/mm^2^), P is the applied load (kg), and d is the average indentation diagonal length (mm). The fracture toughness (K_IC_) of the glass samples was estimated using the half-penny crack model, as given in Equation (2) [36,37,38]:

(2) KIC=0.016(EHv)12(Pc32)

where K_IC_ is the fracture toughness (MPa·m^1/2^), E is the Young’s modulus (GPa), *H_v_* is the Vickers hardness (kg/mm^2^), *P* is the applied load (kg), and c is the crack length (m). The brittleness index (*B*) was calculated using Equation (3):

(3) $$B=\frac{H_{v}}{K_{IC}}$$

where B represents brittleness (µm^−1/2^). Uniaxial compression tests were performed using a universal testing machine (Shimadzu Corporation, Kyoto, Japan) on cylindrical samples (5 mm height × 2 mm diameter) to evaluate compressive strength and fracture behavior under axial loading conditions. Vickers microhardness, fracture toughness, and compressive strength measurements were performed on five independent specimens for each composition (*n* = 5), and the results are reported as mean ± standard deviation.

The elastic properties of the glasses were theoretically estimated using the Makishima–Mackenzie model, which relates mechanical behavior to the packing density of the glass network and the dissociation energy per unit volume of the constituent oxides. Based on these parameters, the Young’s modulus (*E_m_*), bulk modulus (*K_m_*), shear modulus (*G_m_*), and Poisson’s ratio (*σ_m_*) were calculated. The equations used in these calculations are summarized in Table 5 [39,40].

### 2.4. Silver Coating

Silver surface modification was applied to the undoped TB glass and the TBY3 composition, selected as the optimal Y_2_O_3_-doped sample based on its balanced mechanical performance, cytocompatibility, controlled ion-release behavior, and preliminary antibacterial response. TB was chosen as the unmodified reference composition to assess the direct effect of Ag-based surface functionalization on the base borotellurite glass. TBY3 was selected as the representative Y_2_O_3_-modified composition because it provided the most balanced combination of cytocompatibility, mechanical performance, controlled ion release, and antibacterial behavior among the doped glasses. Although TBY1 showed the highest long-term viability and TBY7 exhibited the highest hardness and cell adhesion, TBY3 demonstrated a more favorable overall compromise without the pronounced reduction in compressive strength observed at higher Y_2_O_3_ contents. Therefore, coating TB and TBY3 enabled a direct comparison between the base glass and an optimized Y_2_O_3_-containing composition while keeping the experimental design focused.

A silver-containing sol was prepared via an acid-catalyzed sol–gel route using tetraethyl orthosilicate (TEOS; Merck, Germany) as the silica precursor. TEOS (5 mL) was mixed with ethanol (11 mL) and stirred at 34 °C for 20 min. Separately, AgNO_3_ (Merck, Germany; 51 mg, 0.01 M equivalent) was dissolved in a mixture of ethanol (Merck, Germany; 11 mL), deionized water (3 mL), and HNO_3_ (Merck, Germany; catalyst). The silver precursor solution was added dropwise to the TEOS solution under continuous stirring. The resulting sol was aged at 34 °C for 2 h, then at room temperature for 48 h. Prior to coating, samples were ultrasonically cleaned in acetone, ethanol, and deionized water, and dried at 75 °C. Coatings were deposited by dip-coating, with immersion for 1 min followed by controlled withdrawal and drying at room temperature for 10 min. The process was repeated to obtain a double-layer coating. Thermal consolidation was performed by heating to 250 °C at 2 °C/min, holding for 30 min, and cooling to room temperature. The coated samples were designated as Ag-TB and Ag-TBY3. The presence and distribution of the coating were subsequently confirmed by surface characterization using scanning electron microscopy (SEM).

### 2.5. Ion Release Quantification by ICP-MS

The dissolution behavior of the glass samples and their ion release profiles under physiological-like conditions were evaluated by inductively coupled plasma mass spectrometry (ICP-MS) following immersion in simulated body fluid (SBF). The SBF solution was prepared according to the protocol of Kokubo and Takadama [42]. The ionic composition of the SBF used in this study was as follows: Na^+^, 142.0 mM; K^+^, 5.0 mM; Mg^2+^, 1.5 mM; Ca^2+^, 2.5 mM; Cl^−^, 103.0 mM; HCO_3_^−^, 27.0 mM; HPO_4_^2−^, 1.0 mM; and SO_4_^2−^, 0.5 mM. This composition was selected to approximate the inorganic ionic concentrations of human blood plasma and to assess the in vitro bioactive response of the prepared glasses under physiological-like conditions. Each glass disc was immersed individually in 15 mL of SBF in sealed polypropylene tubes and incubated at 37 °C under static conditions.

Ion release was quantified at 1 and 30 days to assess short- and long-term dissolution behavior. At each time point, the entire immersion medium was collected, and the samples were gently rinsed with 1 mL of deionized water to ensure complete recovery of dissolved species. The rinse solution was combined with the collected SBF, and the final solution was acidified with HNO_3_ to 2% (*v*/*v*) prior to analysis. The concentrations of B, Na, P, Ca, Te, and Y ions released into the solution were determined using ICP-MS ( Agilent Technologies, Santa Clara, CA, USA) with external calibration and internal standard correction to minimize matrix effects. All measurements were performed in triplicate (*n* = 3), and the results are reported in ppb (µg/L).

### 2.6. In Vitro Biological Assessment of Bioactive Glasses

The biological performance of the prepared bioactive glass compositions was evaluated through a combination of in vitro bioactivity, cytocompatibility, surface cell response, degradation behavior, and antibacterial adhesion analyses. In vitro bioactivity was assessed by monitoring appetite-forming ability following immersion in simulated body fluid (SBF; Colin Chemistry, Türkiye). Cytocompatibility was assessed using the Alamar Blue assay, and cell adhesion and spreading behavior on glass surfaces were examined by scanning electron microscopy (SEM; EVO LS 10, Carl Zeiss Microscopy GmbH, Jena, Germany). The in vitro degradation behavior of the samples was determined by weight loss measurements in phosphate-buffered saline (PBS). In addition, antibacterial performance was evaluated by a quantitative viable counting method based on the number of bacteria remaining adhered to the sample surfaces after incubation.

#### 2.6.1. Formation of Hydroxyapatite in SBF

The in vitro bioactivity of the glass compositions was evaluated by immersion in simulated body fluid (SBF) at 37 °C for 14 days, following the protocol described by Kokubo and Takadama [42]. Disc-shaped samples were individually immersed in sealed polyethylene containers containing SBF at a controlled surface area-to-volume ratio. To maintain ion concentrations comparable to physiological conditions, the SBF solution was renewed every 7 days during the immersion period. After 14 days, the samples were removed, gently rinsed with deionized water to eliminate loosely attached residues, and air-dried at room temperature. The formation of apatite-like layers on the sample surfaces was characterized by X-ray diffraction (XRD), as described in Section 2.2, and by field-emission scanning electron microscopy (FESEM). The elemental composition of the surface precipitates was analyzed to determine the Ca/P atomic ratio and to evaluate its proximity to stoichiometric hydroxyapatite. In addition, attenuated total reflectance Fourier-transform infrared (ATR-FTIR) spectroscopy was performed to identify phosphate- and carbonate-related vibrational bands associated with apatite formation.

#### 2.6.2. Cell Viability Test

The cytocompatibility of the bioactive glass samples was evaluated using the MC3T3-E1 murine preosteoblast cell line (ATCC CRL-2593, ATCC, Manassas, VA, USA), which was kindly provided as a gift by Istinye University, Istanbul, Türkiye, and is a well-established in vitro model for bone-related biomaterials. Cells were cultured in α-minimum essential medium (α-MEM) supplemented with 10% fetal bovine serum (FBS; Gibco, Grand Island, NY, USA) and 1% penicillin–streptomycin, and maintained at 37 °C in a humidified incubator with 5% CO_2_.

Prior to cell seeding, glass disc samples were sterilized by immersion in 70% ethanol for 30 min, followed by 20 min of ultraviolet (UV) irradiation on each side. The sterilized samples were then placed individually into 24-well culture plates. Cells were seeded onto the samples at a density of 1 × 10^4^ cells per well. Tissue culture polystyrene (TCPS) without samples was used as the negative control, while a 2 mM ZnSO_4_ solution served as the positive cytotoxic control. Cell viability was assessed after 24 h and 30 days using the Alamar Blue assay. For each measurement, Alamar Blue reagent was added to the culture medium at a final concentration of 10% (*v*/*v*), followed by incubation for 4 h at 37 °C. Fluorescence intensity was measured using a microplate reader at excitation and emission wavelengths of 560 and 590 nm, respectively. Cell viability was expressed as a percentage relative to the negative control (100%). According to ISO 10993-5:2009, samples exhibiting cell viability above 70% were classified as non-cytotoxic [43]. All experiments were performed in triplicate (n = 3), and the results are presented as mean ± standard deviation.

#### 2.6.3. Cell Adhesion and Morphology Analysis

Cell adhesion and spreading morphology were examined using field-emission scanning electron microscopy (FESEM; ZEISS Gemini300, Carl Zeiss Microscopy GmbH, Jena, Germany). Glass discs were sterilized as described above and placed on 24-well plates. MC3T3-E1 cells were seeded at 2 × 10^4^ cells/well and incubated for 24 h to allow initial attachment. After incubation, wells were gently washed twice with PBS to remove non-adherent cells. Adherent cells were fixed in 2.5% glutaraldehyde in 0.1 M sodium cacodylate buffer (pH 7.4) for 1 h at 4 °C. Samples were subsequently dehydrated through a graded ethanol series (50%, 70%, 90%, 100% ×2), dried using the critical-point drying method or hexamethyldisilazane (HMDS) evaporation, sputter-coated with gold-palladium, and examined by FESEM. The percentage of cells adhering to the glass surface, relative to the total number of seeded cells, was quantified by counting cells in representative fields of view (at least 3 fields per sample at ×200 magnification). All experiments were performed in triplicate.

#### 2.6.4. In Vitro Biodegradation

The in vitro degradation behavior of the bioactive glass samples was evaluated in phosphate-buffered saline (PBS) under physiological-like conditions over an immersion period of 1–30 days. Each specimen was immersed individually in PBS and maintained at 37 °C throughout the test period. At the end of each designated immersion interval, the samples were removed from the solution, gently rinsed with deionized water to eliminate loosely attached surface residues, and dried at 75 °C for 40 min. The dried samples were then weighed using an analytical balance with precision of five decimal places.

The degradation behavior was assessed in terms of weight loss (*W_L_*), which was calculated according to Equation (4) [5]:

(4) $$W_{L}(\%)=\left[\frac{\left(W_{i}-W_{d}\right)}{W_{i}}\right]\times 100$$

where *W_i_* represents the weights of the samples before immersion in the phosphate-buffered solution; *W_d_* represents their weights after being stored in PBS on specific days and then dried.

#### 2.6.5. Quantitative Analysis of Bacterial Adhesion (CFU Method)

The antibacterial performance of the bioactive glass samples was assessed by quantifying viable bacteria adhered to the sample surfaces using a colony-forming unit (CFU) assay. Gram-negative *Escherichia coli* (ATCC 11775) and Gram-positive *Staphylococcus aureus* (ATCC 25923) were selected as representative bacterial strains [44,45].

Prior to testing, disc-shaped samples were sterilized by ultraviolet (UV) irradiation and placed individually into 24-well plates. Bacterial suspensions were adjusted to 0.5 McFarland standard and diluted 1:10 in tryptic soy broth (TSB; Merck KGaA, Darmstadt, Germany). Each sample was incubated with the bacterial suspension at 35–37 °C for 24 h under static conditions.

Following incubation, samples were gently rinsed with phosphate-buffered saline (PBS) to remove non-adherent bacteria. The adherent bacteria were then detached from the sample surfaces using a combination of ultrasonication and vortexing. The resulting bacterial suspensions were serially diluted and plated onto tryptic soy agar (TSA; Merck KGaA, Darmstadt, Germany). After incubation for 24 h, colonies were counted on plates containing 30–300 CFU, and the results were expressed as CFU per disc. All experiments were performed in triplicate (n = 3), and the data are reported as mean ± standard deviation.

## 3. Results and Discussion

### 3.1. Structural, Optical, Thermal, and Physical Characterization of the Glasses

#### 3.1.1. X-Ray Diffraction Analysis

**Figure 1:**
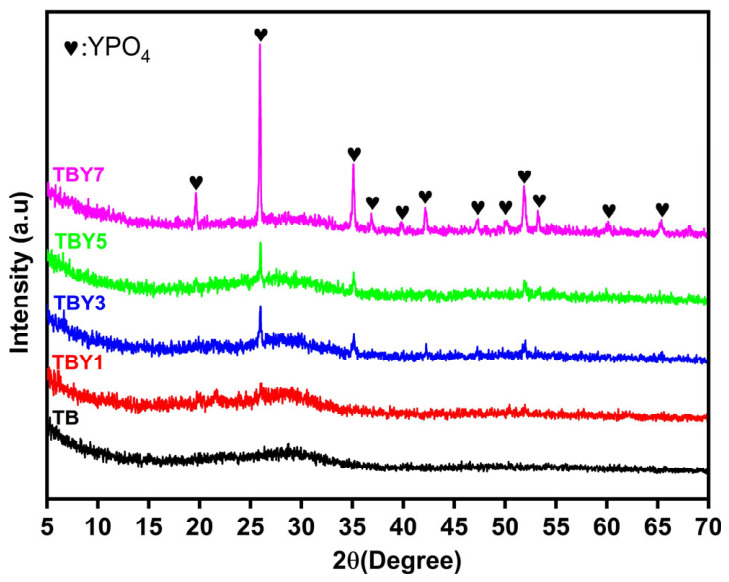
XRD patterns of Y_2_O_3_-doped glass samples.

The structural evolution of the Y_2_O_3_-modified borotellurite glasses was investigated by X-ray diffraction (XRD) to confirm the amorphous nature of the prepared samples and to identify any crystalline phase formation induced by yttrium incorporation. The XRD patterns of the glass samples are presented in Figure 1.

The undoped TB sample exhibited two broad diffuse halos, which are characteristic of an amorphous glass network and indicate the absence of long-range structural ordering [46,47]. This result confirms that the base TeO_2_–B_2_O_3_–P_2_O_5_-containing composition was successfully obtained in a glassy state. With the incorporation of Y_2_O_3_, the glass-forming character of the system was largely preserved; however, weak diffraction peaks began to emerge, indicating the onset of partial crystallization. These peaks were consistent with the xenotime-type YPO_4_ phase (ICDD 01-074-2429), suggesting that yttrium incorporation promoted the formation of phosphate-rich crystalline domains within the predominantly amorphous matrix.

The increasing intensity of these diffraction peaks with increasing Y_2_O_3_ content indicates that crystallization became more pronounced at higher dopant levels. This behavior suggests that Y_2_O_3_ not only modifies the local glass structure but also affects the balance between glass stability and nucleation tendency. The higher ionic field strength of Y^3+^ ions enables them to act as strong coordination centers in the glass network, facilitating interactions with phosphate units and promoting local structural rearrangements favorable for YPO_4_ formation [48,49]. At relatively low Y_2_O_3_ concentrations (1–3 mol.%), these crystalline regions appear to remain limited and dispersed within the amorphous matrix. In contrast, at higher concentrations (5–7 mol.%), the greater density of yttrium-rich centers likely enhances nucleation and crystal growth, leading to the clearer and sharper YPO_4_ reflections observed in the XRD patterns [15,48]. Overall, the XRD results indicate that Y_2_O_3_ incorporation preserves the predominantly glassy nature of the system while progressively increasing its tendency toward partial crystallization at elevated dopant contents.

#### 3.1.2. Fourier-Transform Infrared (FTIR) Analysis

**Figure 2:**
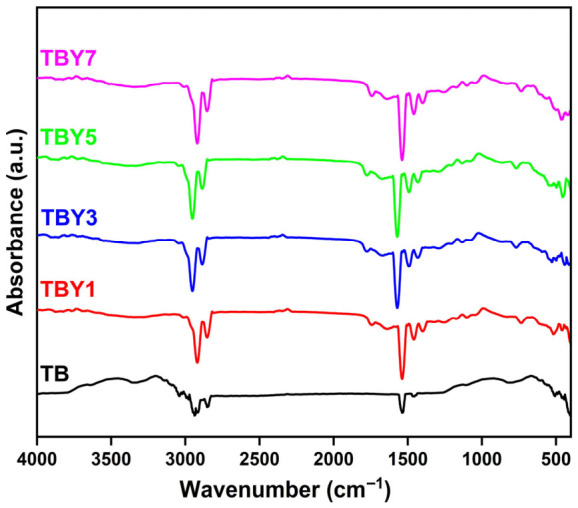
FTIR spectra of Y_2_O_3_-doped borotellurite glass samples.

FTIR spectroscopy was used to investigate the structural changes induced by Y_2_O_3_ incorporation in the borotellurite glass network. The spectra of the prepared samples are shown in Figure 2. All compositions exhibited characteristic absorption bands associated with TeO_2_-, B_2_O_3_-, and P_2_O_5_-based structural units, confirming the multicomponent nature of the glass system and indicating that the principal glass-forming units were retained after Y_2_O_3_ addition.

The spectra displayed several bands in the regions of approximately 2919, 2850, 1746, 1641, 1536, 1460, 1432, 1257, 1096, 1068, 734, 509, and 454 cm^−1^. The weak bands observed near 2850–2920 cm^−1^ are generally attributed to adsorbed water, hydroxyl-containing species, or residual surface contaminants. In contrast, the bands within the 1200–1600 cm^−1^ region are more structurally significant and can be assigned mainly to B–O stretching vibrations of [BO_3_] units, non-bridging B–O bonds, and borate-related ring structures. In particular, the band near 1257 cm^−1^ is associated with boroxole ring vibrations, indicating the presence of borate structural motifs within the glass network [50]. The emergence and increased definition of bands around 1432 and 1460 cm^−1^ after Y_2_O_3_ addition suggest that yttrium modifies the local borate environment and may promote the formation of additional non-bridging oxygens or partial depolymerization within the borate sub-network.

The doublet-like features observed around 1096 and 1068 cm^−1^ likely arise from overlapping vibrations of borate and phosphate units, reflecting the structural heterogeneity expected in a multicomponent glass system. The appearance of these split bands after Y_2_O_3_ incorporation suggests a redistribution of local bonding environments rather than complete network disruption. The band around 734 cm^−1^, which becomes more evident in Y_2_O_3_-containing samples, is attributed to vibrations involving non-bridging oxygen atoms linked to [TeO_3_] trigonal pyramidal units [50,51]. This finding suggests that yttrium affects the local tellurite coordination environment and may favor the formation or enhanced spectral detectability of TeO_3_-related structural species. Finally, the low-wavenumber bands near 509 and 454 cm^−1^ are assigned to Te–O–Te and O–Te–O bending vibrations, with possible contributions from phosphate-related bending modes [51,52,53]. Overall, the FTIR results indicate that Y_2_O_3_ incorporation does not alter the fundamental multicomponent character of the glass network, but it does induce measurable local structural rearrangements in both the borate and tellurite environments.

#### 3.1.3. UV–Vis Optical Analysis

**Figure 3:**
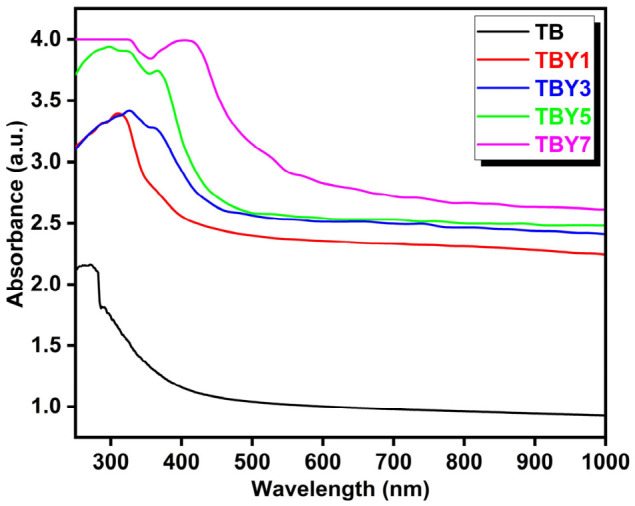
UV–Vis absorption spectra of Y_2_O_3_-doped borotellurite glass samples.

UV–Vis spectroscopy was employed to evaluate the optical response of the Y_2_O_3_-modified borotellurite glass system and to examine the effect of yttrium incorporation on the electronic structure of the glasses. The absorption spectra recorded for the prepared samples are presented in Figure 3. All compositions except TBY7 exhibited broad absorption behavior without a sharp absorption edge, which is characteristic of amorphous materials and is consistent with the XRD results confirming the predominantly glassy nature of the system [47].

**Figure 4:**
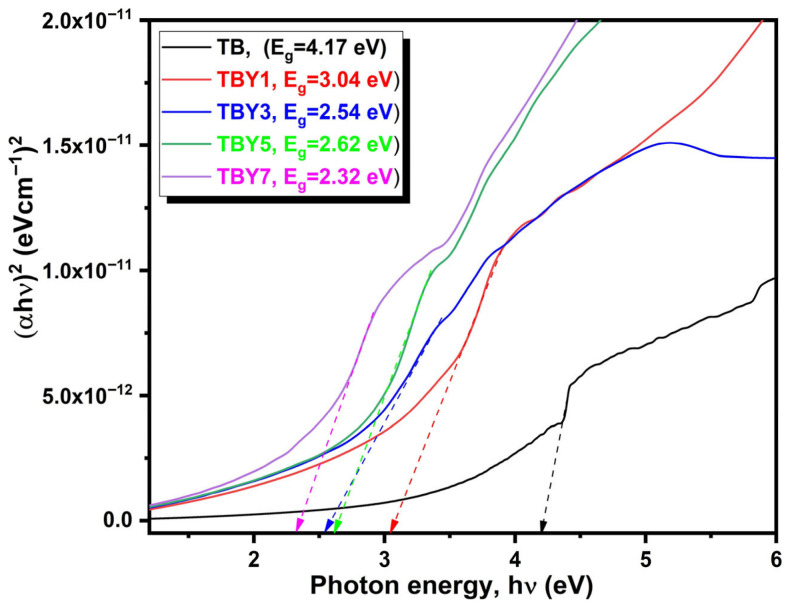
Tauc plots of Y_2_O_3_-doped borotellurite glasses.

A progressive shift of the absorption edge toward longer wavelengths was observed as the Y_2_O_3_ content increased. The absorption wavelength increased from 274 nm for the undoped TB sample to 408 nm for TBY7, indicating a clear red shift in the optical response. This trend suggests that Y_2_O_3_ incorporation alters the local bonding environment and affects the electronic transition behavior of the glass network. Similar red-shift behavior has been reported in rare-earth-modified borate- and phosphate-based glass systems [54,55,56]. The optical band gap values were estimated using the Tauc method, and the corresponding $(\alpha h\nu {)}^{2}$ versus hν plots are shown in Figure 4. The calculated band gap values decreased markedly from 4.17 eV for TB to 2.32 eV for TBY7, demonstrating that yttrium addition significantly narrows the optical band gap.

The reduction in band gap energy can be attributed to local structural rearrangements induced by Y_2_O_3_, which likely promote the formation of defect-related states and non-bridging oxygen-associated electronic levels within the glass network [57,58]. These additional localized states reduce the energy required for electronic excitation and thereby shift the absorption edge toward lower photon energies. In multicomponent amorphous systems, such behavior is also commonly associated with increased structural disorder and the development of band-tail states [59,60,61]. Although surface roughness may contribute to light scattering and broaden the effective absorption edge, the overall trend observed here is consistent with intrinsic compositional modification of the glass structure rather than a purely surface-related effect.

The calculated optical constants are summarized in Table 6. In parallel with the reduction in band gap, the refractive index increased from 2.13 in TB to 2.61 in TBY7. This inverse relationship between band gap and refractive index is well known in oxide glass systems and reflects increased electronic polarizability as the energy separation between valence and conduction bands decreases [62]. The damping coefficient also increased with Y_2_O_3_ content, while the dielectric constant and linear dielectric susceptibility rose from 4.54 to 6.81 and from 0.28 to 0.46, respectively, indicating enhanced polarization capability in the modified glasses. In contrast, the metallization criterion decreased from 0.457 to 0.341, remaining within the range reported for oxide glasses with potential for nonlinear optical behavior [63].

The optical basicity showed a slight decrease from 0.880 to 0.869 with increasing Y_2_O_3_ content. This trend can be explained by the relatively lower basicity contribution of Y_2_O_3_ compared with TeO_2_- and Na_2_O-rich structural environments. As Y_2_O_3_ progressively substitutes for TeO_2_, the average electron donor ability of oxygen ions in the glass decreases slightly, shifting the network toward a comparatively less basic and more electronically stabilized state. Overall, the UV–Vis results demonstrate that Y_2_O_3_ incorporation significantly modifies the optical behavior of borotellurite glasses by narrowing the band gap, increasing refractive index and dielectric response, and altering the electronic polarizability of the network. These findings further support the conclusion that yttrium acts as an effective compositional modifier capable of tuning not only the structural and mechanical properties, but also the optical characteristics of the developed bioactive glass system.

#### 3.1.4. Thermal Properties

**Figure 5:**
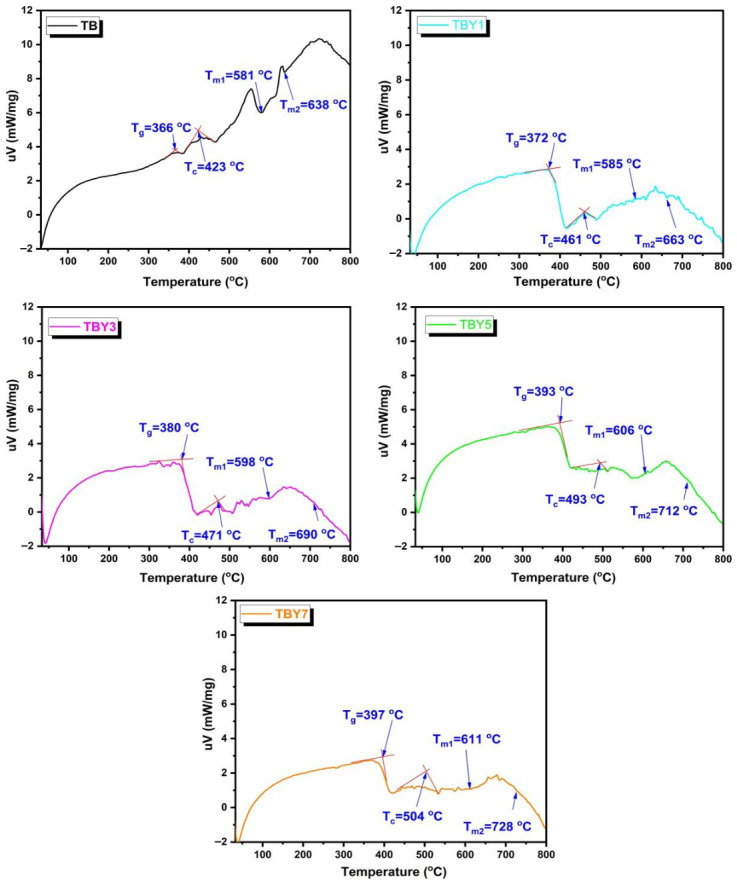
DTA curves of Y_2_O_3_-doped borotellurite glass samples.

The thermal behavior of the Y_2_O_3_-modified borotellurite glasses was evaluated by differential thermal analysis (DTA), and the corresponding thermograms are presented in Figure 5. The characteristic thermal parameters extracted from these curves, including the glass transition temperature (T_g_), crystallization temperature (T_c_), first melting temperature (T_m1_), second melting temperature (T_m2_), and thermal stability window (ΔT = T_c_ − T_g_), are summarized in Table 7.

A clear compositional dependence was observed for all thermal parameters. The glass transition temperature increased progressively from 366 °C in undoped TB glass to 397 °C in TBY7. A similar increasing trend was also recorded for the crystallization temperature, which rose from 423 °C to 504 °C, as well as for the first and second melting temperatures, which increased from 581 °C and 638 °C in TB to 611 °C and 728 °C in TBY7, respectively. Most importantly, the thermal stability window (ΔT) increased markedly from 57 °C in TB to 107 °C in TBY7, indicating that the addition of Y_2_O_3_ substantially improved the glass network’s resistance to devitrification during heating.

The progressive increase in T_g_ with increasing Y_2_O_3_ content indicates that yttrium incorporation enhances the rigidity of the glass network. This behavior can be attributed primarily to the stronger Y–O bond energy (E_Y–O_ = 715 kJ mol^−1^) compared with the Te–O bond (E_Te–O_ = 391 ± 8 kJ mol^−1^) [64]. The substitution of TeO_2_ by Y_2_O_3_ therefore introduces stronger local bonding interactions, which restrict segmental mobility within the glass structure and shift the glass transition to higher temperatures. The simultaneous increase in T_c_ and melting temperatures further supports the conclusion that Y_2_O_3_ acts as a network-stabilizing oxide, thereby improving the overall thermal robustness of the borotellurite system.

The marked widening of the ΔT parameter is particularly important from a processing perspective, as it reflects an increased working range between glass transition and crystallization onset. A larger ΔT value indicates better glass-forming ability and improved thermal processability, reducing the likelihood of uncontrolled crystallization during shaping or thermal treatment [35]. In the present study, the monotonic increase in ΔT with Y_2_O_3_ addition suggests that yttrium not only stiffens the network but also enhances kinetic resistance to crystallization. Similar behavior has been reported in other rare-earth-containing oxide glasses. For example, in the 52B_2_O_3_–12SiO_2_–26Bi_2_O_3_–(10 − x)TiO_2_–xY_2_O_3_ system, increasing Y_2_O_3_ content also led to higher T_g_, T_c_, T_m_, and ΔT values, which were attributed to the formation of stronger Y–O–B linkages and a more thermally stable glass network [65]. The same mechanism is likely operative in the present borotellurite system, where Y_2_O_3_ contributes to increased bond strength and network stabilization.

#### 3.1.5. Physical Properties

**Figure 6:**
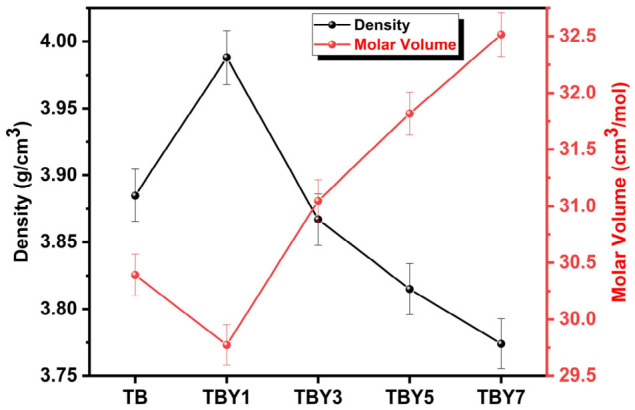
Variation in density and molar volume with Y_2_O_3_ content.

The calculated physical parameters of the Y_2_O_3_-modified borotellurite glasses, including density (*ρ*), atomic packing density (*Vt*), dissociation energy per unit volume (*Gt*), molar volume (*V_M_*, oxygen packing density (*OPD*), molar oxygen volume (*MOV*), average cross-link density (${\bar{n}}_{c}$), and bond density (*n_b_*), are summarized in Table 8. The relationship between density and molar volume is additionally illustrated in Figure 6.

The incorporation of Y_2_O_3_ produced a distinctly non-linear effect on the physical structure of the glasses. The density initially increased from 3.885 g/cm^3^ for the undoped TB composition to 3.988 g/cm^3^ for TBY1, and then gradually decreased to 3.774 g/cm^3^ for TBY7. In contrast, the molar volume followed the opposite trend, decreasing from 30.393 cm^3^/mol in TB to 29.774 cm^3^/mol in TBY1, and then increasing progressively to 32.515 cm^3^/mol in TBY7. This inverse relationship between density and molar volume is characteristic of variations in network packing efficiency and indicates that Y_2_O_3_ exerts a concentration-dependent effect on the structural compactness of the borotellurite glass system [66].

At low Y_2_O_3_ concentration, particularly in TBY1, the simultaneous increase in density and decrease in molar volume suggest that limited yttrium incorporation promotes a more compact structural arrangement. This interpretation is supported by the fact that both *Vt* and OPD reached their highest values in TBY1 (0.584 and 75.905 mol/L, respectively). These trends indicate more efficient atomic and oxygen packing within the glass network at low yttrium levels. Similar densification behavior has been reported in rare-earth-modified oxide glasses and is generally attributed to the relatively high field strength of Y^3+^ ions, which promotes tighter coordination of surrounding oxygen atoms and improved local packing efficiency [66,67]. However, this compaction effect did not persist at higher Y_2_O_3_ contents. Beyond 1 mol.% Y_2_O_3_, density decreased continuously, while molar volume and molar oxygen volume increased. In the present system, this behavior can be explained by two concurrent effects. First, the progressive substitution of TeO_2_ (*ρ* = 5.67 g/cm^3^) by Y_2_O_3_ (*ρ* = 5.01 g/cm^3^) lowers the overall mass contribution per unit volume. Second, the increase in MOV from 13.508 cm^3^/mol in TB to 14.015 cm^3^/mol in TBY7 indicates that the oxygen sub-network becomes progressively more open, suggesting volumetric expansion and reduced packing efficiency at higher yttrium levels [68,69,70]. The accompanying decline in OPD and Vt further supports this interpretation, indicating that although yttrium strengthens local bonding, excessive addition leads to a structurally looser and less efficiently packed network.

Despite this structural opening at higher dopant contents, the dissociation energy per unit volume (G_t_) increased monotonically from 49.600 to 50.909 kJ/cm^3^, while the average cross-link density (${\bar{n}}_{c}$) rose from 2.000 to 2.092 across the series. These results demonstrate that Y_2_O_3_ simultaneously strengthens the average bond network, even as the overall packing efficiency decreases. This behavior is consistent with the high bond dissociation energy of Y–O bonds (715 kJ/mol), which increases the average bond strength of the glass structure. In contrast, the bond density (*n_b_*) increased slightly at low yttrium content and then decreased from 8.658 × 10^22^ ions/cm^3^ in TBY1 to 8.262 × 10^22^ ions/cm^3^ in TBY7, which is consistent with the rise in molar volume and the reduced number of bonds per unit volume in a more open network. Similar dual behavior, namely reduced packing efficiency but increased bond strength, has been reported in tellurite- and borophosphate-based glasses containing rare-earth oxides [70,71].

#### 3.1.6. Morphological Characterization of the Silver Coating

**Figure 7:**
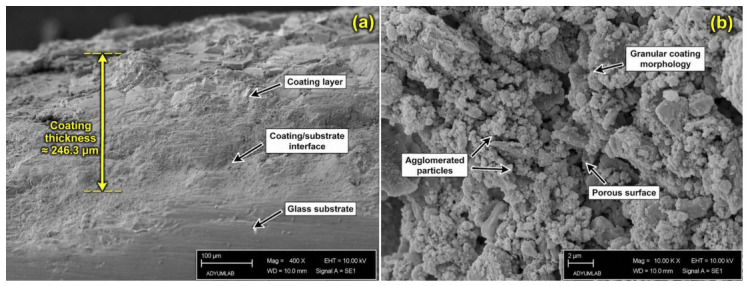
SEM micrographs of the silver-coated sample showing (**a**) the cross-sectional coating thickness and (**b**) the surface morphology of the coating.

SEM images (Figure 7a,b) confirmed the formation of a continuous silver-containing coating on the glass surface. Cross-sectional analysis showed an average coating thickness of approximately 246.28 µm, indicating that the deposited layer was sufficiently developed to act as a potential reservoir for sustained antibacterial ion release. The coating appeared generally compact and continuous, which is important because the antibacterial performance of Ag-containing coatings is strongly associated with coating continuity, surface coverage, and controlled Ag release [28,29,30,31]. However, its relatively high thickness may also influence interfacial stress, adhesion strength, and long-term mechanical stability. In the literature, silver-based surface coatings are widely recognized for their broad-spectrum antibacterial efficacy and their ability to reduce bacterial adhesion and biofilm formation on biomaterial surfaces [29,30,72,73,74]. At the same time, excessively thick coatings may increase the risk of cracking, delamination, or residual stress accumulation, which can compromise structural reliability and coating adhesion [30,31]. Therefore, the present results suggest that the coating was successfully formed and is likely beneficial for antibacterial functionality, but further optimization of coating thickness and interfacial adhesion would be valuable for improving long-term stability and translational potential.

### 3.2. Elastic and Mechanical Properties

The elastic properties of the Y_2_O_3_-modified borotellurite glasses were theoretically estimated using the Makishima–Mackenzie model, and the calculated elastic modulus (*E_m_*), bulk modulus (*K_m_*), shear modulus (*G_m_*), longitudinal modulus (*L_m_*), and Poisson’s ratio (σ_m_) are summarized in Table 9. As shown, the elastic response of the glass system exhibited a composition-dependent trend rather than a monotonic increase with Y_2_O_3_ addition. The elastic modulus increased slightly from 56.416 GPa in the undoped TB glass to 58.131 GPa in TBY1, and then gradually decreased to 56.253 GPa in TBY7. A similar trend was observed for *K_m_*, *G_m_*, and *L_m_*, all of which reached their maximum values at low yttrium content and showed a slight decline with further Y_2_O_3_ incorporation. These results indicate that limited Y_2_O_3_ addition improves the stiffness of the glass network, whereas higher dopant levels do not provide further enhancement in elastic response.

The observed elastic behavior can be explained by considering the dual effect of Y_2_O_3_ on glass structure. In oxide glasses, elastic properties are governed primarily by bond strength and packing efficiency; therefore, both the dissociation energy of the network and the density of atomic packing contribute to the final elastic response [65,66]. In the present study, the initial increase in *E_m_* at TBY1 is consistent with the physical-property data discussed previously, where the highest atomic packing density (*V_t_* = 0.584) and oxygen packing density (*OPD* = 75.905 mol/L) were also recorded for this composition. This suggests that a small amount of Y_2_O_3_ promotes a more compact and mechanically efficient glass network. However, as the Y_2_O_3_ content increased further, the gradual decline in *E_m_*, *K_m_*, *G_m_*, and *L_m_* indicates that the reduction in packing efficiency and the increase in molar volume begin to outweigh the strengthening contribution of Y–O-related bonding. In other words, higher Y_2_O_3_ levels strengthen local bonds but simultaneously generate a more open and less efficiently packed structure, resulting in only limited elastic benefit.

**Figure 8:**
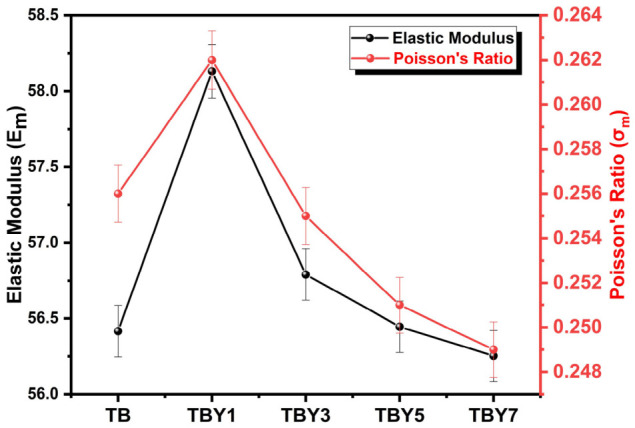
Effect of Y_2_O_3_ addition on the elastic modulus and Poisson’s ratio of borotellurite glasses.

Poisson’s ratio also followed a composition-dependent trend, increasing slightly from 0.256 in TB to 0.262 in TBY1 and then decreasing progressively to 0.249 in TBY7. This behavior further supports the interpretation that low-level Y_2_O_3_ addition improves structural compactness and mechanical balance, whereas higher concentrations induce increasing heterogeneity and structural opening. The relationship between Y_2_O_3_ content, elastic modulus, and Poisson’s ratio is illustrated in Figure 8.

The experimental mechanical properties of the glass series are presented in Table 10. Vickers hardness increased continuously from 2.824 GPa in TB to 4.317 GPa in TBY7, demonstrating that Y_2_O_3_ incorporation markedly enhances resistance to localized deformation. Fracture toughness also improved significantly, increasing from 1.190 MPa·m^1/2^ in TB to 2.289 MPa·m^1/2^ in TBY7. In contrast, the maximum compressive stress showed a non-monotonic trend, reaching its highest value at TBY1 (67.973 MPa) and then decreasing substantially with further Y_2_O_3_ addition, ultimately falling to 22.132 MPa in TBY7. These contrasting trends indicate that Y_2_O_3_ is highly effective in improving local indentation resistance and crack resistance, but does not continuously enhance the bulk load-bearing capacity of the glass under compression.

The progressive increase in hardness can be attributed to the strengthening effect of Y–O-related bonding, together with the monotonic increase in dissociation energy per unit volume (G*_t_*) and average cross-link density (${\bar{n}}_{c}$). These parameters indicate that Y_2_O_3_ reinforces the local bond network and improves its resistance to indentation-induced deformation [70]. The simultaneous increase in fracture toughness and the decrease in brittleness from 2.373 µm^−1/2^ in TB to 1.625 µm^−1/2^ in TBY5 suggest that moderate yttrium incorporation improves resistance to crack initiation and propagation more effectively than it increases brittleness. However, the slight increase in brittleness at TBY7 (1.886 µm^−1/2^), despite the highest hardness in the series, suggests that the network at the highest Y_2_O_3_ content becomes locally harder but less able to dissipate crack-tip stresses, thereby producing a slightly more brittle response under indentation loading.

**Figure 9:**
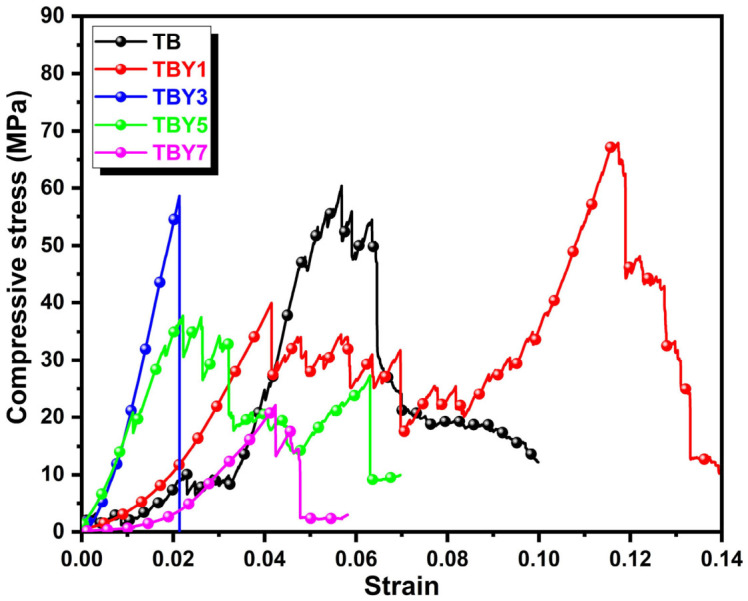
Compressive strengths of Y_2_O_3_-doped borotellurite bioactive glasses.

The compressive behavior followed a different structural logic. Unlike hardness, compressive strength depends not only on local bond strength, but also on volumetric packing efficiency, structural homogeneity, and the ability of the network to distribute stress uniformly under axial loading. The highest compressive strength observed in TBY1 is fully consistent with the highest density and packing density values recorded for this composition, indicating that optimum structural compactness was achieved at low Y_2_O_3_ content. With further Y_2_O_3_ addition, the decrease in density, Vt, and OPD, together with the increase in V_M_ and MOV, indicates the development of a more open and less efficiently packed glass structure. Such structural loosening likely reduces the material’s ability to distribute compressive stress homogeneously, making it more sensitive to local defects and promoting earlier catastrophic failure under bulk loading [69]. This interpretation is also consistent with the stress–strain behavior shown in Figure 9, where compositions containing higher Y_2_O_3_ contents exhibit weaker compressive response.

From a biomedical standpoint, the compressive strength values obtained in this study (22–68 MPa) remain below the typical range reported for cortical bone (100–200 MPa) [75,76]. However, they fall within or near the range often reported for trabecular bone and for bioactive glass materials intended for non-load-bearing or low-load bone defect filling applications [5,77]. Therefore, although the present compositions may not yet be suitable for high load-bearing orthopedic applications, they remain relevant for bone repair scenarios where bioactivity, cytocompatibility, and controlled dissolution are prioritized over high compressive strength.

### 3.3. Assessment of ICP Results

The ion-release behavior of the Y_2_O_3_-modified borotellurite bioactive glasses was evaluated after immersion in simulated body fluid (SBF) for 1 and 30 days, and the corresponding ICP-MS results are summarized in Table 11. These measurements were used to assess both the early-stage dissolution behavior and the longer-term release profile of biologically relevant ions from the glass network.

After 1 day, the Y_2_O_3_-containing compositions released markedly higher amounts of Na, P, and Ca than the undoped TB glass, indicating enhanced early-stage ionic exchange. In contrast, Te release decreased substantially in all doped samples relative to TB, suggesting that Y_2_O_3_ suppresses the initial release of tellurium-containing species. After 30 days, ion concentrations increased in all groups, as expected from continued dissolution; however, the undoped TB sample still exhibited the highest B and Te release, whereas the Y_2_O_3_-containing glasses maintained considerably lower tellurium concentrations. Among the doped compositions, TBY3 showed the highest Y release (0.751 ppb), while Na and Ca release remained relatively high across all groups.

These findings indicate that Y_2_O_3_ exerts a dual effect on the dissolution behavior of the borotellurite glass system. It appears to facilitate the release of biologically relevant ions such as Na, Ca, and P during the early stage, while simultaneously stabilizing the network against excessive long-term tellurium release through stronger Y–O interactions. The overall release profile also suggests controlled degradation rather than rapid network breakdown, likely due to the gradual formation of a surface reaction layer during immersion. Therefore, Y_2_O_3_ modification improves the biological suitability of the glass not only by altering composition, but also by regulating ion release in a more favorable manner.

### 3.4. Biocompatibility and In Vitro Analyses

#### 3.4.1. Assessment of Hydroxyapatite Formation

**Figure 10:**
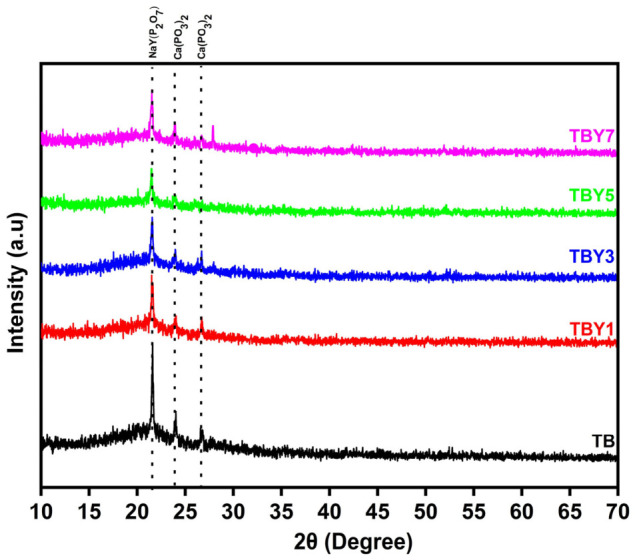
XRD spectra of Y_2_O_3_-doped bioactive glasses after 14 days of immersion in SBF.

The in vitro bioactivity of the prepared glasses was evaluated by XRD after 14 days of immersion in simulated body fluid (SBF), and the corresponding diffraction patterns are shown in Figure 10. Following SBF incubation, the glass surface exhibited diffraction features consistent with sodium yttrium diphosphate (NaY(P_2_O_7_), ICDD 01-087-1913) and calcium catenate phosphate (Ca(PO_3_)_2_, ICDD 01-079-0700), indicating the formation of phosphate-rich reaction products on the glass surface. These findings confirm that the Y_2_O_3_-modified borotellurite glasses retained their ability to undergo surface reactions under physiological-like conditions, which is a key indicator of in vitro bioactivity.

The formation of such calcium- and phosphate-containing surface phases suggests that dissolution of the glass network was followed by reprecipitation processes at the glass–solution interface. This behavior is consistent with the established bioactivity mechanism of bioactive glasses, in which ionic exchange and network dissolution promote the development of a surface reaction layer that subsequently acts as a precursor for apatite-like mineralization [42,78]. Although the detected phases were not identified directly as stoichiometric hydroxyapatite, their phosphate-rich nature indicates that the glass surfaces were actively participating in mineral deposition. Similar intermediate calcium phosphate or pyrophosphate-type phases have been reported in multicomponent bioactive glass systems during the early stages of SBF mineralization, prior to the maturation of a more apatite-like layer [42,78]. Therefore, the XRD results suggest that Y_2_O_3_ incorporation does not suppress the in vitro bioactive response of the borotellurite glass system and may still support the formation of biologically relevant mineralized surface products.

#### 3.4.2. Assessment of Cell Viability (Alamar Blue) Results

**Figure 11:**
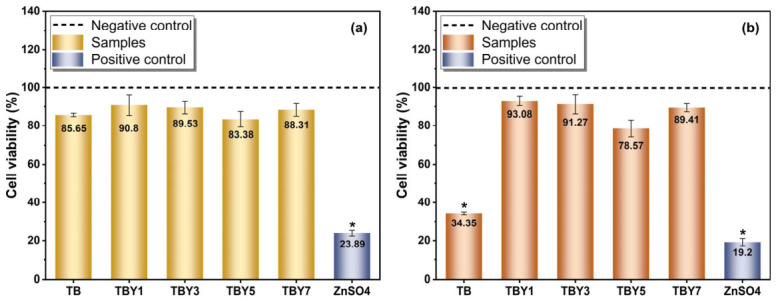
Viability (%) of MC3T3-E1 cells incubated with Y_2_O_3_-doped bioactive glass samples for (**a**) 24 h and (**b**) 30 days. The dashed black line represents the viability of the control group without sample. * Statistically significant difference compared with the negative control group (*p* < 0.05).

The biological response of the prepared bioactive glass samples toward osteoblast-like MC3T3-E1 cells was evaluated in vitro using the Alamar Blue viability assay after 24 h and 30 days of incubation. The negative control group without any sample was taken as the 100% viability reference, whereas ZnSO_4_ (2 mM) was used as the positive cytotoxic control. The Cell viability (%) of MC3T3-E1 cells cultured with Y_2_O_3_-doped bioactive glass samples for 1 and 30 days is shown in Figure 11.

At 24 h, all glass compositions maintained relatively high cell viability, ranging from 83.38 ± 4.05% to 90.80 ± 5.46%, indicating that none of the tested glasses induced acute cytotoxicity during the early exposure period. In contrast, the positive control showed markedly reduced viability (23.89 ± 1.47%), confirming the sensitivity of the assay. After 30 days, the differences among the compositions became much more pronounced. The undoped TB sample showed a substantial reduction in cell viability to 34.35 ± 0.71%, whereas all Y_2_O_3_-containing glasses remained above the 70% threshold defined by ISO 10993-5:2009 for non-cytotoxic materials [43]. Among them, TBY1 exhibited the highest long-term viability (93.08 ± 2.58%), followed by TBY3 (91.27 ± 5.12%), TBY7 (89.41 ± 2.14%), and TBY5 (78.57 ± 4.39%). These results clearly show that Y_2_O_3_ incorporation markedly improved long-term cytocompatibility compared with the undoped borotellurite glass.

When evaluated collectively, the results suggest that yttrium addition exerts a composition-dependent effect on osteoblast viability. The superior long-term performance of the Y_2_O_3_-containing samples, particularly TBY1, may be explained by the stabilizing effect of Y^3+^ on the glass network. As discussed in the structural and ICP-MS results, Y_2_O_3_ incorporation reduced excessive long-term tellurium release while maintaining controlled dissolution of biologically relevant ions. This is particularly important because prolonged release of tellurite species has been associated with oxidative stress and mitochondrial dysfunction in mammalian cells [32]. In the present study, the severe long-term viability loss observed in the undoped TB sample is consistent with this mechanism, whereas the significantly improved viability of the Y_2_O_3_-modified glasses supports the idea that yttrium acts not only as a mechanical modifier but also as a biocompatibility-enabling network stabilizer.

The slight decline in viability observed at higher Y_2_O_3_ contents relative to TBY1 may reflect changes in dissolution kinetics, ionic concentration, or local pH conditions associated with increasing dopant levels. Such behavior is not unusual in multicomponent bioactive glasses, where cellular response depends strongly on achieving a balance between beneficial ion release and excessive local chemical perturbation. Previous studies have similarly reported that Y_2_O_3_-containing glass and ceramic systems can display acceptable cytocompatibility when the yttrium content remains within an appropriate compositional window [20,21,22]. Therefore, the present results indicate that careful optimization of Y_2_O_3_ concentration is critical, with low-to-moderate yttrium incorporation providing the most favorable balance between structural stability and biological performance. Overall, the Alamar Blue results confirm that the Y_2_O_3_-modified borotellurite glasses are substantially more suitable for bone-related applications than the undoped composition, particularly under long-term exposure conditions relevant to implantable biomaterials.

#### 3.4.3. Osteoblast Adhesion and Morphological Evaluation

**Figure 12:**
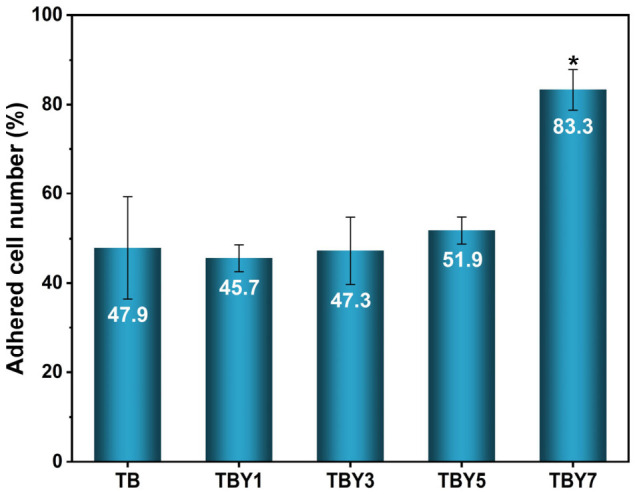
Number of cells adhering to the surface of Y_2_O_3_-doped bioactive glasses (%). *: Statistically significant difference compared to the TB reference sample (*p* < 0.05).

**Figure 13:**
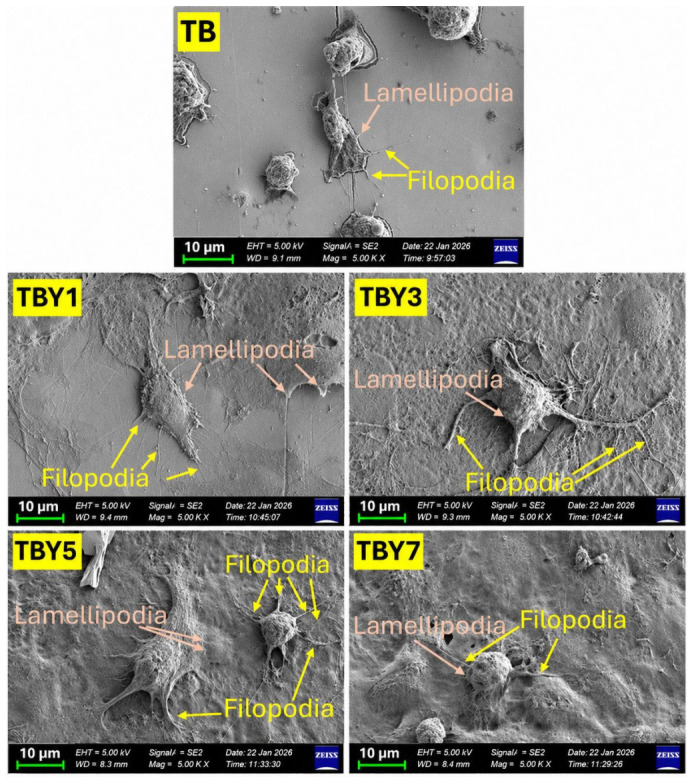
FESEM images of cells adhering to the surface of Y_2_O_3_-doped bioactive glasses.

The adhesion behavior of MC3T3-E1 osteoblast-like cells on the surfaces of the Y_2_O_3_-doped bioactive glass samples was evaluated, and the corresponding results are presented in Figure 12. Representative FESEM images illustrating the morphology of cells attached to the sample surfaces are shown in Figure 13.

The results demonstrate that increasing Y_2_O_3_ content enhances cell–surface interactions and promotes more effective osteoblast attachment to the bioactive glass surfaces. As shown in Figure 12, the adhesion levels of TB, TBY1, and TBY3 were comparable, while a moderate increase was observed for TBY5. A pronounced rise in cell adhesion was recorded for TBY7, which exhibited the highest adhesion value (83.33 ± 4.55%) and a statistically significant difference compared with the TB reference. These findings indicate that high yttrium incorporation positively influences the surface properties of the glass in a manner favorable for early osteoblast attachment.

Cell adhesion is one of the most critical parameters governing the early biological performance of biomaterials, as it directly affects subsequent cell spreading, proliferation, and differentiation [2,79,80,81]. In the present study, low yttrium contents did not substantially alter the adhesion response; however, increasing the Y_2_O_3_ concentration led to a marked enhancement in cell attachment. The strong adhesion observed for TBY7 suggests that yttrium incorporation may induce favorable chemical and physicochemical modifications at the glass surface, such as improved protein adsorption capacity or increased surface energy. It is well established that biomaterial–cell interactions are mediated by adsorbed proteins and surface chemistry, which regulate integrin-mediated attachment and focal adhesion formation [82,83].

The FESEM observations in Figure 13 further support the quantitative adhesion data. On the TB surface, cells appeared relatively sparse and less spread, indicating weaker anchorage. In contrast, the Y_2_O_3_-containing samples, particularly TBY5 and TBY7, exhibited more extensive spreading and stronger attachment to the substrate. The FESEM images also reveal the presence of lamellipodia and filopodia, which are well-recognized indicators of active cytoskeletal organization and dynamic cell–surface interaction [84,85]. These protrusive structures are associated with actin remodeling and integrin engagement at the cell–material interface, and their presence reflects a biologically favorable surface for osteoblast adhesion [81,84].

The improved adhesion observed at higher Y_2_O_3_ contents is consistent with previous reports on bioactive glasses and rare-earth-modified biomaterials, in which enhanced surface reactivity and controlled ionic dissolution were shown to support osteoblast attachment and early cellular response [2,4,14,80]. In particular, ionic dissolution products released from bioactive glasses can modulate signaling pathways involved in osteogenic activity [14], while surface topography and nanoscale features may further influence integrin binding and cytoskeletal organization [86]. In the present system, the superior adhesion behavior of TBY7 likely reflects a synergistic effect of optimized surface chemistry, regulated ion release, and improved interfacial energy conditions induced by Y_2_O_3_ incorporation.

Overall, the combined quantitative adhesion data and FESEM-based morphological observations demonstrate that Y_2_O_3_ content significantly affects the early osteoblast response to borotellurite bioactive glasses. While low yttrium contents had only a limited influence on adhesion, higher concentrations particularly in the TBY7 composition substantially improved both the number of adhered cells and their spreading behavior.

#### 3.4.4. In Vitro Degradation Behavior

**Figure 14:**
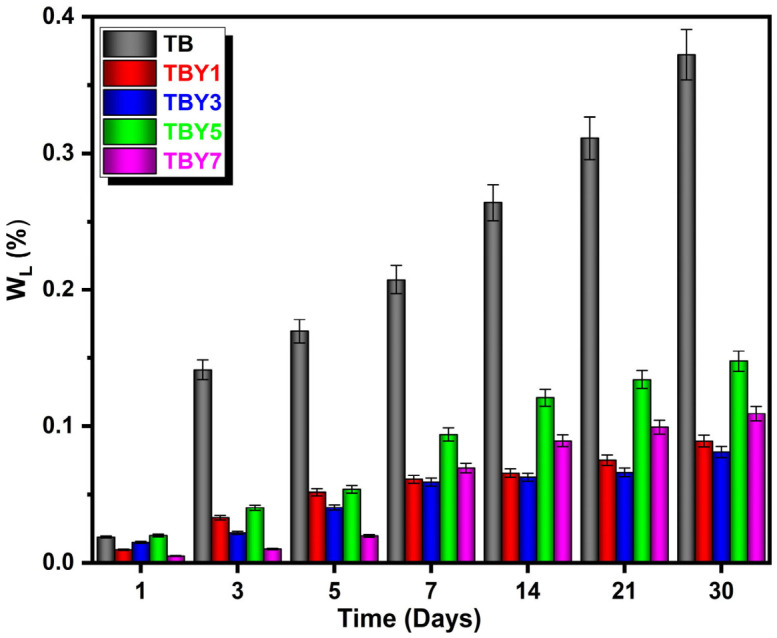
Time-dependent weight loss of Y_2_O_3_-doped borotellurite glasses immersed in PBS.

The biodegradation behavior of the Y_2_O_3_-doped bioactive glasses was evaluated by immersing the samples in PBS for predetermined time intervals and calculating the corresponding percentage weight loss. The time-dependent weight loss profiles are presented in Figure 14.

As shown in Figure 14, the weight loss increased progressively with immersion time for all compositions, indicating continuous interaction between the glass surface and the surrounding solution. However, the undoped TB sample consistently exhibited the highest degradation level throughout the test period, demonstrating a faster dissolution rate than the Y_2_O_3_-containing glasses. Although weight loss also increased over time in the doped groups, their overall degradation remained lower than that of the unmodified composition.

These results suggest that Y_2_O_3_ incorporation improves the chemical durability of the borotellurite glass system. This effect can be attributed to the strengthening of cation–oxygen interactions and the resulting increase in network stability, which reduces the susceptibility of the glass to dissolution in aqueous media [18,87,88,89]. Similar findings have been reported for rare-earth-modified glass systems, where the introduction of rare-earth oxides enhanced chemical resistance by increasing network rigidity and cross-linking strength [90]. Therefore, the present results indicate that Y_2_O_3_ acts as an effective stabilizing modifier that moderates biodegradation while preserving the reactive character of the bioactive glass.

#### 3.4.5. Antibacterial Performance and Bacterial Adhesion

The antibacterial performance of the Y_2_O_3_-doped borotellurite bioactive glasses was evaluated by viable bacterial counting after adhesion of *Escherichia coli* ATCC 11775 and *Staphylococcus aureus* ATCC 25923 on the sample surfaces. The corresponding live-count results are summarized in Table 12.

**Figure 15:**
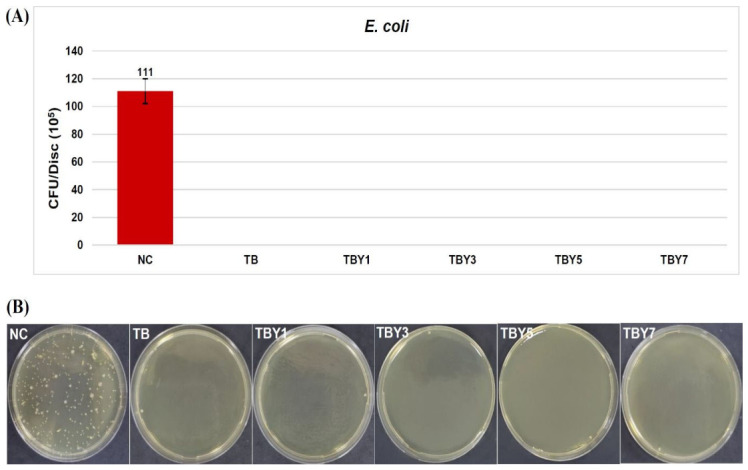
Live count results for *E. coli* ATCC 11775 adhering to the surface of Y_2_O_3_-doped glass samples (**A**) and corresponding Petri dish images (**B**). NC: negative control.

For *E. coli*, no viable bacterial adhesion was detected on either the undoped TB glass or any of the Y_2_O_3_-containing bioactive glass samples, whereas the negative control glass of identical surface area exhibited a bacterial count of 111 × 10^5^ CFU/disk, confirming that the assay conditions were valid and that the absence of detectable adhesion on the tested glasses reflected a genuine antibacterial response rather than experimental failure. These results are presented in Figure 15.

**Figure 16:**
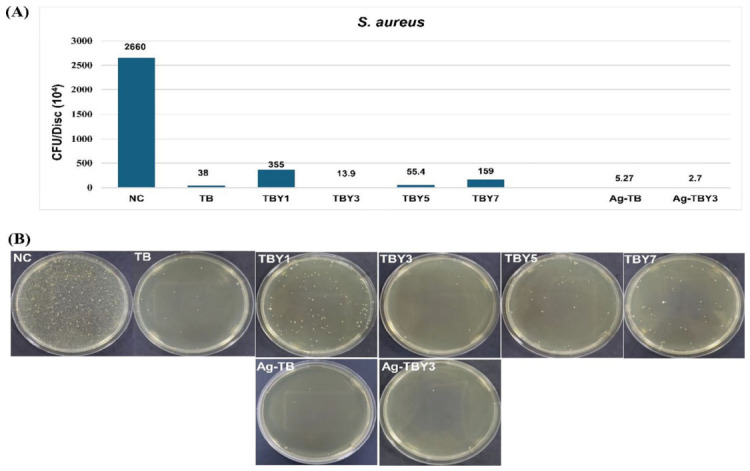
Live count results for *S. aureus* ATCC 25923 adhering to the surface of Y_2_O_3_-doped glass samples (**A**) and corresponding Petri dish images (**B**). NC: negative control.

For *S. aureus*, measurable bacterial adhesion was detected on all uncoated glass samples, although the extent of adhesion strongly depended on composition. Among the Y_2_O_3_-doped groups, TBY3 exhibited the lowest bacterial count (13.9 ± 2.4 × 10^4^ CFU/disk), indicating the strongest anti-adhesive effect within the doped series. Relative to the undoped TB sample, this corresponded to an adhesion inhibition of 63.4%, while inhibition relative to the negative control reached approximately 99.5%. After silver coating of the TBY3 composition, the bacterial count decreased further to 2.7 ± 0.5 × 10^4^ CFU/disk, corresponding to an additional 80.6% reduction compared with uncoated TBY3. In comparison with TB and the negative control, the adhesion inhibition achieved by Ag-TBY3 reached 92.9% and 99.9%, respectively. These results are shown in Figure 16.

The antibacterial trends observed in this study can be interpreted in light of both the ion-release behavior and the surface chemistry of the glasses. ICP-MS analysis confirmed the release of tellurium-containing species from the borotellurite compositions, and the absence of detectable *E. coli* adhesion may be associated with the known antimicrobial effect of tellurite species. Tellurite ions are highly toxic to many bacterial cells because they disrupt thiol metabolism, induce oxidative stress, and interfere with essential cellular redox processes. Gram-negative bacteria can be particularly sensitive to these effects, which may explain the complete suppression of detectable *E. coli* adhesion on all tested glass surfaces [32]. At the same time, the composition-dependent differences observed for *S. aureus* suggest that Y_2_O_3_ incorporation influences antibacterial behavior not only through dissolution products, but also through its effect on surface structure and interfacial interactions. In this respect, the superior performance of TBY3 suggests that an intermediate Y_2_O_3_ level provides the most favorable balance between antibacterial ion release and surface characteristics that limit bacterial colonization.

The marked improvement after silver coating is fully consistent with the well-established broad-spectrum antimicrobial activity of silver and its derivatives. Silver ions are known to act through multiple simultaneous mechanisms, including membrane disruption, enzyme inactivation, reactive oxygen species generation, and binding to nucleic acids and proteins, which collectively reduce the likelihood of resistance development compared with conventional single-target antibiotics [28,29,91,92]. Recent studies have also shown that integrating silver into biomaterial surfaces effectively reduces bacterial adhesion and biofilm formation, thereby lowering the risk of implant-associated infection [30,31,72,73,74]. In the present work, coating the most promising Y_2_O_3_-doped composition (TBY3) with silver further enhanced the anti-adhesive response against *S. aureus*, confirming a clear synergistic benefit of combining compositional optimization with antibacterial surface functionalization. Overall, these findings demonstrate that Y_2_O_3_-doped borotellurite bioactive glasses possess intrinsic antibacterial potential, while silver coating provides an additional and highly effective strategy for strengthening infection-resistant functionality.

### 3.5. Limitations and Future Perspectives

The present study provides an initial in vitro evaluation of Y_2_O_3_-modified borotellurite bioactive glasses through cytocompatibility, osteoblast adhesion, SBF bioactivity, ion-release, degradation, and antibacterial analyses. However, osteogenic differentiation was not directly confirmed by ALP activity, matrix mineralization, or osteogenic gene-expression analysis. Therefore, future studies should include ALP activity, Alizarin Red S mineralization assays, and osteogenic markers such as RUNX2, ALP, COL1A1, OCN, and OPN to more clearly verify osteoinductive potential.

Further work should also assess long-term coating stability, degradation under dynamic conditions, and in vivo bone-defect performance to better establish practical applicability. In addition, XPS analysis should be performed to clarify surface chemical states and coating–glass interfacial chemistry. Post-melting XRF analysis was performed as a semi-quantitative screening method during the revision process; however, the data were not included in the manuscript because they do not represent absolute final stoichiometric compositions for this multicomponent borotellurite–phosphate glass system. Fully validated bulk compositional verification by ICP-OES or ICP-MS after complete glass digestion is also needed to confirm the final oxide composition more accurately.

## 4. Conclusions

This study demonstrated that Y_2_O_3_ incorporation effectively modifies the structural, thermal, mechanical, biological, and antibacterial behavior of borotellurite bioactive glasses. Y_2_O_3_ increased thermal stability, hardness, and fracture toughness, while low-level yttrium addition provided the highest compressive strength. All Y_2_O_3_-containing glasses maintained cell viability above the ISO 10993-5 non-cytotoxicity threshold after 30 days, indicating improved long-term cytocompatibility compared with the undoped glass. The doped glasses also supported osteoblast adhesion, retained in vitro bioactivity after SBF immersion, and exhibited controlled degradation behavior. Among the doped compositions, TBY3 showed the most favorable overall antibacterial response, and Ag-based surface coating further enhanced its activity against *S. aureus*. Overall, these findings suggest that Y_2_O_3_-modified borotellurite glasses, particularly when combined with Ag surface functionalization, are promising candidates for bone tissue engineering applications requiring cytocompatibility, bioactivity, and antibacterial functionality.

## Figures and Tables

**Table 1:** General characteristics of starting materials.

Material Names	Chemical Formula	Molar Masses (g/mol)	Densities (g/cm^3^)	Melting Temperatures (°C)
Tellurium dioxide	TeO_2_	159.6	5.67	733
Orthoboric acid	H_3_BO_3_	69.63	1.44	170.9
Sodium carbonate	Na_2_CO_3_	105.98	2.54	851
Calcium oxide	CaO	56.07	3.34	2572
Phosphorus pentoxide	P_2_O_5_	141.94	2.39	340
Yttrium oxide	Y_2_O_3_	225.81	5.01	2410

**Table 2:** Prepared glass compositions (mol.%).

Sample Codes	TeO_2_	B_2_O_3_	Na_2_O	CaO	P_2_O_5_	Y_2_O_3_
TB	45	10	20	10	15	0
TBY1	44	10	20	10	15	1
TBY3	42	10	20	10	15	3
TBY5	40	10	20	10	15	5
TBY7	38	10	20	10	15	7

**Table 3:** Formulas used in calculating physical parameters.

Calculated Feature	Equation	Explanations
Density (*ρ*) (gr/cm^3^)	$\rho =\frac{m_{h}}{m_{h}-m_{s}}\times \rho_{s}$	*m_h_*, weight of the sample in air *m_s_*, weight of the sample in liquid
Molar Volume (*V_m_*) (cm^3^/mol)	$V_{m}=\frac{M_{T}}{\rho }$	*M_T_*, total molecular weight of the multi-component glass system
Average cross-linking density (*ñ_c_*)	${ñ}_{c}=\frac{\sum_{i}x_{i}{\left(n_{c}\right)}_{i}{\left(N_{C}\right)}_{i}}{\sum_{i}x_{i}{\left(N_{c}\right)}_{i}}$	_xi_, molar fraction of each component *n_c_*, cross-link per cation *N_c_*, Number of cations per glass formula unit
Number of links (*n_b_*)	$n_{b}=\frac{N_{A}}{V_{M}}\sum_{i}{\left(n_{f}x\right)}_{i}$	*N_A_*, Avagadro’s number *n_f_*, coordination number of cations *x*, molar fraction of each oxide component *i*, oxide component
Molar oxygen volume (*MOV*)	$MOV=\left(\sum \frac{x_{i}M_{i}}{\rho }\right)\left(\frac{1}{\sum x_{i}n_{i}}\right)$	*M_i_*, molecular weight *n_i_*, number of oxygen atoms in each oxide component
Oxygen packing density (*OPD*)	$OPD=1000C\left(\frac{\rho }{M}\right)$	*C*, number of oxygen atoms per compound
Atomic packing density (*V*_t_)	$V_{t}=\frac{\sum f_{i}V_{i}}{\sum \frac{f_{i}M_{i}}{\rho }}$	*f*_i_, molar fraction of the i oxidant
Ionic volume (*V_i_*)	$V_{i}=\frac{4}{3}\pi N_{A}\left(xr_{A}^{3}+yr_{B}^{3}\right)$	*r_A_* ve r_B_ ionic radius
Dissociation energy (*G_t_*)	$G_{t}=\sum_{i}x_{i}G_{i}$	The G_i_ values were obtained from data reported by Inaba et al. (1999) [34]

**Table 4:** Equations used in calculating the optical bandpass and other optical constants.

Calculated Feature	Equation	Explanations
Optical band gap (*E_g_*)	(αh_ν_)^2^ = A(h_ν_ − E_g_)	*A*, constant value *h_υ_*, photon energy *α*, absorption coefficient *E_g_*, bandgap energy
Absorption coefficient (*α*(*λ*))	α(λ)= 2.303 $\frac{A}{d}$	
Refractive index (*n*)	$\frac{n^{2}-1}{n^{2}+2}=1-\sqrt{\frac{E_{opt}}{20}}$	
Damping coefficient (*k*)	$k=\frac{\alpha \lambda }{4\pi }$	
Metallization criteria (*M*)	$M=1-\left[\frac{n^{2}-1}{n^{2}+2}\right]=\sqrt{\frac{E_{opt}}{20}}$	
Dielectric constant (*ε*)	$\varepsilon =n^{2}$	
Optical dielectric constant (*ε_opt_*)	$\varepsilon_{opt}=\varepsilon -1=n^{2}-1$	
Dielectric constant (*χ*)	$\chi =\frac{\epsilon -1}{4\pi }$	

**Table 5:** Elastic properties calculated using the Makishima-Mackenzie Model [39,40,41].

Calculated Properties	Equations	Descriptions
Elastic modulus (GPa)	$E_{m}=2V_{t}G_{t}$	V_t_: atomic packing density G_t_: dissociation energy (It is given in Table 3.)
Shear modulus (GPa)	$G_{m}=\frac{3E_{m}K_{m}}{9K_{m}-E_{m}}$	
Bulk modulus (GPa)	$K_{m}=1.2V_{t}E_{m}$	
Longitudinal Modulus (GPa)	$L_{m}=K_{m}+\left(\frac{4G_{m}}{3}\right)$	
Poisson’s ratio	$\sigma_{m}=\left(\frac{E_{m}}{2G_{m}}\right)-1$	
Microhardness (GPa)	$H=\frac{\left(1-2\sigma_{m}\right)E_{m}}{6\left(1+\sigma_{m}\right)}$	

**Table 6:** Optical band gap and related optical constants of Y_2_O_3_-doped borotellurite glasses.

	Sample Codes				
Optical Parameters	TB	TBY1	TBY3	TBY5	TBY7
Bandgap (Ev)	4.17	3.04	2.54	2.62	2.32
Absorption wavelength (nm)	274	315	331	303	408
Absorption coefficient (×10^2^ m^−1^)	1.45	1.42	1.25	1.75	1.65
Refractive index	2.13	2.39	2.53	2.51	2.61
Extinction coefficient (×10^−8^)	3.2	3.6	3.3	4.2	5.4
Metallization criterion	0.457	0.389	0.356	0.362	0.341
Optical electronegativity	1.12	0.84	0.68	0.70	0.62
Optical basicity (Λ)	0.88	0.876	0.873	0.871	0.869
Dielectric constant	4.54	5.71	6.40	6.30	6.81
Optical dielectric constant	3.54	4.71	5.40	5.30	5.81
Linear electrical or dielectric susceptibility	0.28	0.38	0.43	0.42	0.46

**Table 7:** Thermal properties of Y_2_O_3_-doped borotellurite glasses.

Sample Codes	T_g_ (°C)	T_c_ (°C)	T_m1_ (°C)	T_m2_ (°C)	ΔT (°C)
TB	366	423	581	638	57
TBY1	372	461	585	663	89
TBY3	380	471	598	690	91
TBY5	393	493	606	712	100
TBY7	397	504	611	728	107

**Table 8:** Physical parameters of Y_2_O_3_-doped borotellurite glass samples.

Sample Codes	*ρ* (g/cm^3^)	*V_t_* (cm^3^/mol)	*G_t_* (kJ/cm^3^)	*V_M_* (cm^3^/mol)	*OPD* (mol/L)	*MOV* (cm^3^/mol)	*ñ_c_*	*n_b_* (×10^22^) (ions/cm^3^)
TB	3.885	0.569	49.600	30.393	74.03	13.508	2.000	8.422
TBY1	3.988	0.584	49.787	29.774	75.905	13.174	2.014	8.658
TBY3	3.867	0.566	50.161	31.048	73.434	13.618	2.041	8.419
TBY5	3.815	0.558	50.535	31.819	72.285	13.834	2.067	8.329
TBY7	3.774	0.552	50.909	32.515	71.351	14.015	2.092	8.262

**Table 9:** Elastic properties of Y_2_O_3_-doped borotellurite glass samples.

Sample Codes	TB	TBY1	TBY3	TBY5	TBY7
Elastic modulus (*E_m_*) (GPa)	56.416	58.131	56.791	56.445	56.253
Bulk modulus (*K_m_*) (GPa)	38.502	40.724	38.578	37.828	37.294
Shear modulus (*G_m_*) (GPa)	22.463	23.029	22.632	22.555	22.526
Longitudinal Modulus (*L_m_*) (GPa)	68.452	71.430	68.754	67.901	67.329
Poisson’s ratio (*σ_m_*)	0.256	0.262	0.255	0.251	0.249

**Table 10:** Mechanical properties of Y_2_O_3_-doped borotellurite bioactive glasses.

Sample Codes	Hardness (GPa)	Fracture Toughness (MPa·m^1/2^)	Brittleness (µm^−1/2^)	Max. Compressive Stress (MPa)
TB	2.824	1.190	2.373	60.374
TBY1	3.305	1.694	1.951	67.973
TBY3	3.476	1.822	1.908	58.647
TBY5	3.680	2.265	1.625	37.761
TBY7	4.317	2.289	1.886	22.132

**Table 11:** Ion concentrations (ppb) released from Y_2_O_3_-doped borotellurite bioactive glass samples after 1 and 30 days of immersion in SBF.

Sample Codes	Immersion Time	B	Na	P	Ca	Te	Y
TB	1 day	11.52	63833	1394	3274	5513	0
TBY1		1.99	200296	15517	14570	527	0.150
TBY3		1.68	176995	13462	12894	782	0.223
TBY5		1.48	136674	8141	8983	806	0.109
TBY7		1.62	141105	9337	10411	760	0.181
TB	30 days	303.34	161935	27234	10398	12715	0.000
TBY1		4.61	180647	11549	12437	1315	0.366
TBY3		3.74	156606	9656	10985	1826	0.751
TBY5		4.77	174198	16856	11762	1921	0.243
TBY7		3.45	187380	12991	13180	1627	0.519

**Table 12:** Live count results of test bacteria adhering to the surface of Y_2_O_3_-doped bioactive glass samples with and without silver coating.

Sample Codes	CFU/Disk	
	*E. coli* (×10^5^)	*S. aureus* (×10^4^)
NC (glass)	111 ± 9	2660 ± 135.3
TB	ND	38 ± 7.5
TBY1	ND	355 ± 32.1
TBY3	ND	13.9 ± 2.4
TBY5	ND	55.4 ± 6.1
TBY7	ND	159 ± 47.2
Ag-TBY	-	5.27 ± 0.82
Ag-TBY3	-	2.7 ± 0.5

## Data Availability

The original contributions presented in the study are included in the article; further inquiries can be directed to the corresponding author.

## Abbreviations

The following abbreviations are used in this manuscript:

MC3T3-E1	Murine preosteoblast cell line
PBS	Phosphate-buffered saline
SBF	Simulated body fluid
TCPS	Tissue culture polystyrene
TSB	Tryptic soy broth
TSA	Tryptic soy agar
CFU	Colony-forming unit

## References

[1] Campana V., Milano G., Pagano E., Barba M., Cicione C., Salonna G., Lattanzi W., Logroscino G. (2014). Bone substitutes in orthopaedic surgery: From basic science to clinical practice. J. Mater. Sci. Mater. Med..

[2] Hench L.L., Polak J.M. (2002). Third-generation biomedical materials. Science.

[3] Boccaccini A.R., Brauer D.S., Hupa L. (2016). Bioactive Glasses: Fundamentals, Technology and Applications.

[4] Jones J.R. (2013). Review of bioactive glass: From Hench to hybrids. Acta Biomater..

[5] Fu Q., Rahaman M.N., Fu H., Liu X. (2010). Silicate, borosilicate, and borate bioactive glass scaffolds with controllable degradation rate for bone tissue engineering applications. I. Preparation and in vitro degradation. J. Biomed. Mater. Res. Part A.

[6] Rahaman M. (2014). Bioactive ceramics and glasses for tissue engineering. Tissue Engineering Using Ceramics and Polymers.

[7] Christie J.K., Malik J., Tilocca A. (2011). Bioactive glasses as potential radioisotope vectors for in situ cancer therapy: Investigating the structural effects of yttrium. Phys. Chem. Chem. Phys..

[8] Costerton J.W., Stewart P.S., Greenberg E.P. (1999). Bacterial biofilms: A common cause of persistent infections. Science.

[9] Arnaudov M., Dimitrov V., Dimitriev Y., Markova L. (1982). Infrared-spectral investigation of tellurites. Mater. Res. Bull..

[10] Sabadel J., Armand P., Cachau-Herreillat D., Baldeck P., Doclot O., Ibanez A., Philippot E. (1997). Structural and nonlinear optical characterizations of tellurium oxide-based glasses: TeO_2_–BaO–TiO_2_. J. Solid State Chem..

[11] Miola M., Massera J., Cochis A., Kumar A., Rimondini L., Vernè E. (2021). Tellurium: A new active element for innovative multifunctional bioactive glasses. Mater. Sci. Eng. C.

[12] Baino F., Fiume E., Miola M., Verné E. (2018). Bioactive sol-gel glasses: Processing, properties, and applications. Int. J. Appl. Ceram. Technol..

[13] Gerhardt L.-C., Boccaccini A.R. (2010). Bioactive glass and glass-ceramic scaffolds for bone tissue engineering. Materials.

[14] Hoppe A., Güldal N.S., Boccaccini A.R. (2011). A review of the biological response to ionic dissolution products from bioactive glasses and glass-ceramics. Biomaterials.

[15] Elkhoshkhany N., Marzouk S., El-Sherbiny M., Ibrahim H., Burtan-Gwizdala B., Alqahtani M.S., Hussien K.I., Reben M., Yousef E.S. (2022). Investigation of structural, physical, and attenuation parameters of glass: TeO_2_-Bi_2_O_3_-B_2_O_3_-TiO_2_-RE_2_O_3_ (RE: La, Ce, Sm, Er, and Yb), and applications thereof. Materials.

[16] Shelby J.E. (2020). Introduction to Glass Science and Technology.

[17] Sayyed M., Abdo M., Ali H.E., Sadeq M. (2022). Impact of Y_2_O_3_ on the structural, optical, radiation shielding, and ligand field parameters of transparent borate glass containing constant CrO_3_ and high Na_2_O contents. Ceram. Int..

[18] Mahdy E.A., Ibrahim S. (2012). Influence of Y_2_O_3_ on the structure and properties of calcium magnesium aluminosilicate glasses. J. Mol. Struct..

[19] Christie J.K., Tilocca A. (2012). Integrating biological activity into radioisotope vectors: Molecular dynamics models of yttrium-doped bioactive glasses. J. Mater. Chem..

[20] Albulym O., Kaygili O., Hussien M.S., Zahran H., Kilany M., Darwish R., Bulut N., Alshahrie A., Yahia I. (2021). Synthesis and characterization of yttrium-doped hydroxyapatite nanoparticles and their potential antimicrobial activity. J. Biomater. Tissue Eng..

[21] Nathanael A.J., Mangalaraj D., Hong S., Masuda Y. (2011). Synthesis and in-depth analysis of highly ordered yttrium doped hydroxyapatite nanorods prepared by hydrothermal method and its mechanical analysis. Mater. Charact..

[22] Zhang K., Zhang B., Huang C., Gao S., Li B., Cao R., Cheng J., Li R., Yu Z., Xie X. (2019). Biocompatibility and antibacterial properties of pure titanium surfaces coated with yttrium-doped hydroxyapatite. J. Mech. Behav. Biomed. Mater..

[23] Rohitha D.S., Rajeshwari D., Prasad A., Lakshmi A.M., Rao P.V., Madaboosi N., Özcan M., Prasad P.S. (2026). Engineering Bioactivity through Calcination in Phosphate-Based Mesoporous Glass–Ceramic Nanoparticles for Bone Regeneration. J. Alloys Compd..

[24] Prasad A., Sowjanya V., Manuel J., Rao P.V., Madaboosi N., Prasad P.S. (2025). Silver-Doped Bioactive spherical Glass Ceramic Nanoparticles with Enhanced Osteogenic and Antibacterial Properties for Bone Tissue Engineering. Colloids Surf. A Physicochem. Eng. Asp..

[25] Zimmerli W., Trampuz A., Ochsner P.E. (2004). Prosthetic-joint infections. N. Engl. J. Med..

[26] Bozic K.J., Ries M.D. (2005). The impact of infection after total hip arthroplasty on hospital and surgeon resource utilization. J. Bone Jt. Surg..

[27] Donlan R.M., Costerton J.W. (2002). Biofilms: Survival mechanisms of clinically relevant microorganisms. Clin. Microbiol. Rev..

[28] Feng Q.L., Wu J., Chen G.-Q., Cui F.-Z., Kim T., Kim J. (2000). A mechanistic study of the antibacterial effect of silver ions on Escherichia coli and Staphylococcus aureus. J. Biomed. Mater. Res..

[29] Jung W.K., Koo H.C., Kim K.W., Shin S., Kim S.H., Park Y.H. (2008). Antibacterial activity and mechanism of action of the silver ion in Staphylococcus aureus and Escherichia coli. Appl. Environ. Microbiol..

[30] Mokabber T., Cao H., Norouzi N., Van Rijn P., Pei Y. (2020). Antimicrobial electrodeposited silver-containing calcium phosphate coatings. ACS Appl. Mater. Interfaces.

[31] Morimoto T., Hirata H., Eto S., Hashimoto A., Kii S., Kobayashi T., Tsukamoto M., Yoshihara T., Toda Y., Mawatari M. (2022). Development of silver-containing hydroxyapatite-coated antimicrobial implants for orthopaedic and spinal surgery. Medicina.

[32] Chasteen T.G., Fuentes D.E., Tantaleán J.C., Vásquez C.C. (2009). Tellurite: History, oxidative stress, and molecular mechanisms of resistance. FEMS Microbiol. Rev..

[33] Acikgoz A., Kavun Y., Fidan M., Yorulmaz N. (2026). Nd_2_O_3_ Tailored B_2_O_3_–TeO_2_–Al_2_O_3_–Na_2_O Glasses: Correlating Composition with Structural, Optical, Elastic, and Gamma Shielding Behaviors. J. Alloys Compd..

[34] Inaba S., Fujino S., Morinaga K. (1999). Young’s modulus and compositional parameters of oxide glasses. J. Am. Ceram. Soc..

[35] Saad M., Poulain M. (1987). Glass forming ability criterion. Mater. Sci. Forum.

[36] Aktas B., Yalcin S., Albaskara M., Aytar E., Ceyhan G., Turhan Z.Ş. (2022). Effect of Er_2_O_3_ on structural, mechanical, and optical properties of Al_2_O_3_-Na_2_O-B_2_O_3_-SiO_2_ glass. J. Non-Cryst. Solids.

[37] Anstis G., Chantikul P., Lawn B.R., Marshall D. (1981). A critical evaluation of indentation techniques for measuring fracture toughness: I, direct crack measurements. J. Am. Ceram. Soc..

[38] Aktas B., Albaskara M., Yalcin S., Dogru K. (2017). Mechanical properties of soda-lime-silica glasses with variable peanut shell contents. Acta Phys. Pol. A.

[39] Makishima A., Mackenzie J.D. (1973). Direct calculation of Young’s moidulus of glass. J. Non-Cryst. Solids.

[40] Makishima A., Mackenzie J.D. (1975). Calculation of bulk modulus, shear modulus and Poisson’s ratio of glass. J. Non-Cryst. Solids.

[41] Ulas E.O., Acikgoz A., Aktas B., Kavun Y. (2025). Influence of B_2_O_3_ incorporation on the structural, mechanical and radiation shielding properties of TeO2 Based bioglasses. Appl. Radiat. Isot..

[42] Kokubo T., Takadama H. (2006). How useful is SBF in predicting in vivo bone bioactivity?. Biomaterials.

[43] (2009). Biological Evaluation of Medical Devices—Part 5: Tests for in Vitro Cytotoxicity.

[44] Aktas B., Das R., Aktas H.G., Uyar E., Yalcin S., Ergin B., Celik Z., Ulas E.O. (2025). Additive manufacturing of TiO_2_-doped 3Y-ZrO_2_ ceramics via DLP-3D printing for dental implant applications: Enhanced mechanical strength, biocompatibility, and antibacterial performance. J. Alloys Compd..

[45] Aktas B., Das R., Acıkgoz A., Ulas E.O., Demircan G., Uyar E., Celik Z., Ergin B., Aktas H.G., Yalcin S. (2025). Fabrication of CaSiO_3_-doped 3Y-ZrO_2_ ceramics via DLP 3D printing: Structural, mechanical, and biological evaluation. J. Alloys Compd..

[46] Acikgoz A., Demircan G., Yılmaz D., Aktas B., Yalcin S., Yorulmaz N. (2022). Structural, mechanical, radiation shielding properties and albedo parameters of alumina borate glasses: Role of CeO_2_ and Er_2_O_3_. Mater. Sci. Eng. B.

[47] Solak B.B., Aktas B., Yilmaz D., Kalecik S., Yalcin S., Acikgoz A., Demircan G. (2024). Exploring the radiation shielding properties of B_2_O_3_-PbO-TeO_2_-CeO_2_-WO_3_ glasses: A comprehensive study on structural, mechanical, gamma, and neutron attenuation characteristics. Mater. Chem. Phys..

[48] Kaur G., Kumar M., Arora A., Pandey O., Singh K. (2011). Influence of Y_2_O_3_ on structural and optical properties of SiO_2_–BaO–ZnO–xB_2_O_3_–(10−x) Y_2_O_3_ glasses and glass ceramics. J. Non-Cryst. Solids.

[49] Quintas A., Caurant D., Majérus O., Dussossoy J.-L., Charpentier T. (2008). Effect of changing the rare earth cation type on the structure and crystallisation behaviour of an aluminoborosilicate glass. Phys. Chem. Glas.-Eur. J. Glass Sci. Technol. Part B.

[50] Mansour E. (2012). FTIR spectra of pseudo-binary sodium borate glasses containing TeO_2_. J. Mol. Struct..

[51] Rada S., Culea E., Rada M., Pascuta P., Maties V. (2009). Structural and electronic properties of tellurite glasses. J. Mater. Sci..

[52] Kaur A., Khanna A., Sathe V.G., Gonzalez F., Ortiz B. (2013). Optical, thermal, and structural properties of Nb_2_O_5_–TeO_2_ and WO_3_–TeO_2_ glasses. Phase Transit..

[53] El-Meliegy E., Farag M., Knowles J. (2016). Dissolution and drug release profiles of phosphate glasses doped with high valency oxides. J. Mater. Sci. Mater. Med..

[54] Rao S.L.S., Ramadevudu G., Shareefuddin M., Hameed A., Chary M.N., Rao M.L. (2012). Optical properties of alkaline earth borate glasses. Int. J. Eng. Sci. Technol..

[55] Rasul S.Y., Aktas B., Rustum S.S., Yalcin S., Fahad A., Acikgoz A., Ceyhan G. (2025). Role of HfO_2_ on Modifying the structural, optical, elastic, and mechanical features of boro tellurite glasses. Opt. Mater..

[56] Saudi H.A. (2014). UV–visible and infrared absorption spectra of lead boro-phosphate glasses containing lithium oxide. Sop. Trans. Phys. Chem..

[57] Aktas B., Acikgoz A., Yilmaz D., Yalcin S., Dogru K., Yorulmaz N. (2022). The role of TeO_2_ insertion on the radiation shielding, structural and physical properties of borosilicate glasses. J. Nucl. Mater..

[58] Shakeri M., Rezvani M. (2011). Optical band gap and spectroscopic study of lithium alumino silicate glass containing Y^3+^ ions. Spectrochim. Acta Part A Mol. Biomol. Spectrosc..

[59] Barbi S., Mugoni C., Montorsi M., Affatigato M., Gatto C., Siligardi C. (2018). Structural and optical properties of cerium oxide doped barium bismuth borate glasses. J. Non-Cryst. Solids.

[60] Fujiwara H. (2007). Spectroscopic Ellipsometry: Principles and Applications.

[61] Stagg B.J., Charalampopoulos T.T. (1991). Surface-roughness effects on the determination of optical properties of materials by the reflection method. Appl. Opt..

[62] Hubbezoglu I., Akaoglu B., Dogan A., Keskin S., Bolayir G., Özçelik S., Dogan O.M. (2008). Effect of bleaching on color change and refractive index of dental composite resins. Dent. Mater. J..

[63] Umar S., Halimah M., Chan K., Latif A. (2017). Physical, structural and optical properties of erbium doped rice husk silicate borotellurite (Er-doped RHSBT) glasses. J. Non-Cryst. Solids.

[64] Arafat A., Samad S.A., Wadge M.D., Islam M.T., Lewis A.L., Barney E.R., Ahmed I. (2020). Thermal and crystallization kinetics of yttrium-doped phosphate-based glasses. Int. J. Appl. Glass Sci..

[65] Zheng T., Li M., Ma Y., Jiang H. (2024). Kinetic analysis of the crystallization of Y_2_O_3_ and La_2_O_3_ doped Li_2_O–Al_2_O_3_–SiO_2_ glass. RSC Adv..

[66] Shaaban K.S., Al-Baradi A.M., Wahab E.A. (2022). The impact of Y_2_O_3_ on physical and optical characteristics, polarizability, optical basicity, and dispersion parameters of B_2_O_3_–SiO_2_–Bi_2_O_3_–TiO_2_ glasses. Silicon.

[67] Thabit H.A., Mhareb M., Abdulmalik D., Al-Fakih A.M., Alajerami Y., Hashim S. (2023). Exploring the properties of Dy_2_O_3_–Y_2_O_3_ Co-activated telluro-borate glass: Structural, physical, optical, thermal, and mechanical properties. Heliyon.

[68] Milanova M., Kostov K., Iordanova R., Aleksandrov L., Yordanova A., Mineva T. (2019). Local structure, connectivity and physical properties of glasses in the B_2_O_3_-Bi_2_O_3_-La_2_O_3_-WO_3_ system. J. Non-Cryst. Solids.

[69] Nidzam N.N.S., Kamari H.M., Sukari M.S.M., Alauddin F.A.M., Laoding H., Azlan M., Hann S.W., Al-Hada N.M., Boukhris I. (2022). Comparison study of elastic, physical and structural properties for strontium oxide and manganese oxide in borotellurite glasses for high strength glass application. J. Inorg. Organomet. Polym. Mater..

[70] Li X., Wang Y., Yang P., Han T., Shi X., He K., Zu C. (2022). Effect of Y_2_O_3_/La_2_O_3_ on structure and mechanical properties of Li_2_O–Al_2_O_3_–SiO_2_ glass. J. Non-Cryst. Solids.

[71] Negm H.H., Abdo M., Sadeq M. (2023). Impact of Y2O3 on structural, mechanical and nonlinear optical properties of CrO_3_-Na_2_O-B_2_O_3_ glasses. Optik.

[72] Elbasuney S., El-Sayyad G.S., Radwan S.M., Correa-Duarte M.A. (2022). Antimicrobial, and antibiofilm activities of silver doped hydroxyapatite: A novel bioceramic material for dental filling. J. Inorg. Organomet. Polym. Mater..

[73] Liao Y., Wang X., Chen P., Zhong Q., Gong J. (2025). Antimicrobial mechanisms of metal-based nanomaterials. Int. J. Nanomed..

[74] Escobar M., Turner A.B., Sort J., Nogués C., Pellicer E., Blanquer A., Trobos M. (2025). Cytocompatibility and antimicrobial properties of silver nanoparticle-decorated hydroxyapatite-coated TiMoNbTa alloy. Surf. Interfaces.

[75] Javidan M., Wang K., Moazen M. (2021). Biomechanical studies of human diaphyseal tibia fracture fixation. Computational Modelling of Biomechanics and Biotribology in the Musculoskeletal System.

[76] Reilly D.T., Burstein A.H. (1975). The elastic and ultimate properties of compact bone tissue. J. Biomech..

[77] Cui X., Huang C., Huang W., Wang D., Pan H., Rahaman M.N. (2019). Structural Characteristics of a Family of Bioactive Glasses Formed by Replacing Varying Amounts of SiO_2_ in 45S5 Glass with B_2_O_3_. SSRN. https://ssrn.com/abstract=3407085.

[78] Hench L.L., Splinter R.J., Allen W.C., Greenlee T. (1971). Bonding mechanisms at the interface of ceramic prosthetic materials. J. Biomed. Mater. Res..

[79] Hench L.L. (2006). The story of Bioglass^®^. J. Mater. Sci. Mater. Med..

[80] Baino F., Hamzehlou S., Kargozar S. (2018). Bioactive glasses: Where are we and where are we going?. J. Funct. Biomater..

[81] Anselme K. (2000). Osteoblast adhesion on biomaterials. Biomaterials.

[82] Wilson C.J., Clegg R.E., Leavesley D.I., Pearcy M.J. (2005). Mediation of biomaterial–cell interactions by adsorbed proteins: A review. Tissue Eng..

[83] Keselowsky B.G., Collard D.M., García A.J. (2004). Surface chemistry modulates focal adhesion composition and signaling through changes in integrin binding. Biomaterials.

[84] Mattila P.K., Lappalainen P. (2008). Filopodia: Molecular architecture and cellular functions. Nat. Rev. Mol. Cell Biol..

[85] Ridley A.J. (2011). Life at the leading edge. Cell.

[86] Dalby M.J., Gadegaard N., Oreffo R.O. (2014). Harnessing nanotopography and integrin–matrix interactions to influence stem cell fate. Nat. Mater..

[87] Saleem Q.M., Abo-Mosallam H., Mahdy E.A., Aly K., Ebrahium M.M. (2025). Impact of Y_2_O_3_ on structure, physico-chemical and mechanical properties of new SnO-B_2_O_3_-P_2_O_5_ glasses for radioactive waste immobilization. Ceram. Int..

[88] Singh K., Bala I., Kumar V. (2009). Structural, optical and bioactive properties of calcium borosilicate glasses. Ceram. Int..

[89] Singh S., Kalia G., Singh K. (2015). Effect of intermediate oxide (Y_2_O_3_) on thermal, structural and optical properties of lithium borosilicate glasses. J. Mol. Struct..

[90] Salman S., Salama S., Mahdy E.A. (2019). Crystallization characteristics and properties of lithium germanosilicate glass-ceramics doped with some rare earth oxides. Bol. Soc. Esp. Cerám. Vidr..

[91] Frei A., Verderosa A.D., Elliott A.G., Zuegg J., Blaskovich M.A. (2023). Metals to combat antimicrobial resistance. Nat. Rev. Chem..

[92] Li W.-R., Xie X.-B., Shi Q.-S., Zeng H.-Y., Ou-Yang Y.-S., Chen Y.-B. (2010). Antibacterial activity and mechanism of silver nanoparticles on *Escherichia coli*. Appl. Microbiol. Biotechnol..

